# Longitudinal Analysis of Infant Stool Bacteria Communities Before and After Acute Febrile Malaria and Artemether-Lumefantrine Treatment

**DOI:** 10.1093/infdis/jiy740

**Published:** 2018-12-24

**Authors:** Rabindra K Mandal, Rosie J Crane, James A Berkley, Wilson Gumbi, Juliana Wambua, Joyce Mwongeli Ngoi, Francis M Ndungu, Nathan W Schmidt

**Affiliations:** 1 Department of Microbiology and Immunology, University of Louisville, Kentucky; 2 KEMRI Wellcome Trust Research Programme, Kilifi, Kenya; 3 Centre for Tropical Medicine and Global Health, Nuffield Department of Medicine Research Building, University of Oxford, United Kingdom

**Keywords:** antibiotics, artemether/lumefantrine, infants, malaria, microbiota

## Abstract

**Background:**

Gut microbiota were recently shown to impact malaria disease progression and outcome, and prior studies have shown that *Plasmodium* infections increase the likelihood of enteric bacteria causing systemic infections. Currently, it is not known whether *Plasmodium* infection impacts human gut microbiota as a prelude to bacteremia or whether antimalarials affect gut microbiota. Our goal was to determine to what degree *Plasmodium* infections and antimalarial treatment affect human gut microbiota.

**Methods:**

One hundred Kenyan infants underwent active surveillance for malaria from birth to 10 months of age. Each malaria episode was treated with artemether-lumefantrine (AL). Any other treatments, including antibiotics, were recorded. Stool samples were collected on an approximately biweekly basis. Ten children were selected on the basis of stool samples having been collected before (n = 27) or after (n = 17) a malaria episode and without antibiotics having been administered between collections. These samples were subjected to 16S ribosomal ribonucleic acid gene (V3–V4 region) sequencing.

**Results:**

Bacterial community network analysis revealed no obvious differences in the before and after malaria/AL samples, which was consistent with no difference in alpha and beta diversity and taxonomic analysis at the family and genus level with one exception. At the sequence variant (SV) level, akin to bacterial species, only 1 of the top 100 SVs was significantly different. In addition, predicted metagenome analysis revealed no significant difference in metagenomic capacity between before and after malaria/AL samples. The number of malaria episodes, 1 versus 2, explained significant variation in gut microbiota composition of the infants.

**Conclusions:**

In-depth bioinformatics analysis of stool bacteria has revealed for the first time that human malaria episode/AL treatment have minimal effects on gut microbiota in Kenyan infants.

## Teaser

Although malaria-induced dysbiosis of gut microbiota has been postulated to contribute towards increased susceptibility to Non-typhoid *Salmonella* infections, this report demonstrates that *Plasmodium* infections and oral treatment with antimalarial drugs do not alter stool bacteria populations in Kenyan infants.

Almost half of the world population is at risk of malaria, an infectious disease caused by apicomplexan protozoan parasites of the genus *Plasmodium* [1]. Recent publications have demonstrated the impact of gut microbiota, the complex community of microorganisms that live in the gastrointestinal tract, on malaria disease progression and outcome. Yilmaz et al [2] reported that *Escherichia coli* O86:B7 expresses Galα1-3Galβ1-4GlcNAc-R (α-gal) carbohydrates that can elicit the production of anti-α-gal antibodies that cross-react with α-gal-expressing *Plasmodium* sporozoites, providing protection against the initial liver stage infection. We have shown that gut microbiota modulates the magnitude and kinetics of blood stage rodent *Plasmodium* species parasitemia and subsequent severity of malaria in mice [3]. Likewise, stool microbiota composition in humans is associated with the prospective risk of *Plasmodium falciparum* infection [4]. Rodent *Plasmodium* species can also modulate intestinal tissue and gut bacteria populations; however, this outcome appears to be dependent on the combination of mouse strain and *Plasmodium* species [5, 6].

*Plasmodium* infections in humans increase susceptibility of enteric bacteria progressing to bacteremia, including nontyphoid *Salmonella* [7], and it is well established that gut microbiota dysbiosis increases susceptibility to *Salmonella* infections [8]. Consistent with these observations, *Plasmodium yoelii nigeriensis*-induced gut bacteria dysbiosis increased susceptibility of mice to nontyphoid *Salmonella* infection [6]. More importantly, it remains unknown whether *Plasmodium* infection alters human gut microbiota as a potential prelude to the progression of enteric bacteria to bacteremia. Moreover, although oral antibiotics cause gut microbiota dysbiosis [9, 10], which increases susceptibility to numerous diseases [11–15], it is not known whether oral treatment with antimalarial drugs also impacts gut microbiota. Therefore, the objective of the current study was to assess to what degree *Plasmodium* infection, or antimalarial treatment, in humans induces gut bacteria dysbiosis that may contribute to enteric bacteria progressing to systemic infections.

## METHODS

### Study Site and Sample Collection

Full details are provided in Supplementary Text. In brief, 100 infants in Kilifi County, Kenya, were subject to continuous active and passive case detection for fever and other health parameters from within 14 days of birth until 9 months of age (cohort principal investigator R.J.C.). Febrile participants with positive rapid diagnostic test (RDT) and/or slide microscopy were immediately started on a 3-day oral course of artemether-lumefantrine (AL). Antibiotic courses were also prospectively recorded. Stool samples were collected at home every 1–3 weeks. Samples were selected for analysis on the basis of having been collected before or fewer than 18 days after a malaria diagnosis, with no antibiotics having been administered during the period in-between the “before” and “after” stool samples. Deoxyribonucleic acid was extracted from these selected stool samples and subjected to 16S ribosomal ribonucleic acid (rRNA) gene sequence analysis of the V3–V4 hypervariable region according to the Illumina 16S metagenomic sequencing library preparation protocol (Illumina). Reads underwent quality control to remove phiX reads and filter chimeric sequences followed by multiple downstream analyses of high-quality sequences using QIIME2(https://qiime2.org/). Statistical analysis for alpha and beta diversity were performed using linear mixed-effect models at 33 000 sequencing reads depth per sample. Metagenomic capacity was predicted using Piphillin (http://secondgenome.com/solutions/resources/data-analysis-tools/piphillin/), and statistical analysis were performed using *t* test and DESeq2 implemented inside RNA-seq 2G online tool (http://52.90.192.24:3838/rnaseq2g/). Variation and significance level of covariates were determined using “envfit” function implemented in “vegan” (http://cc.oulu.fi/~jarioksa/softhelp/vegan/html/envfit.html).

## RESULTS

### Participants, Stool Samples, and Sequencing

**Figure 1.**
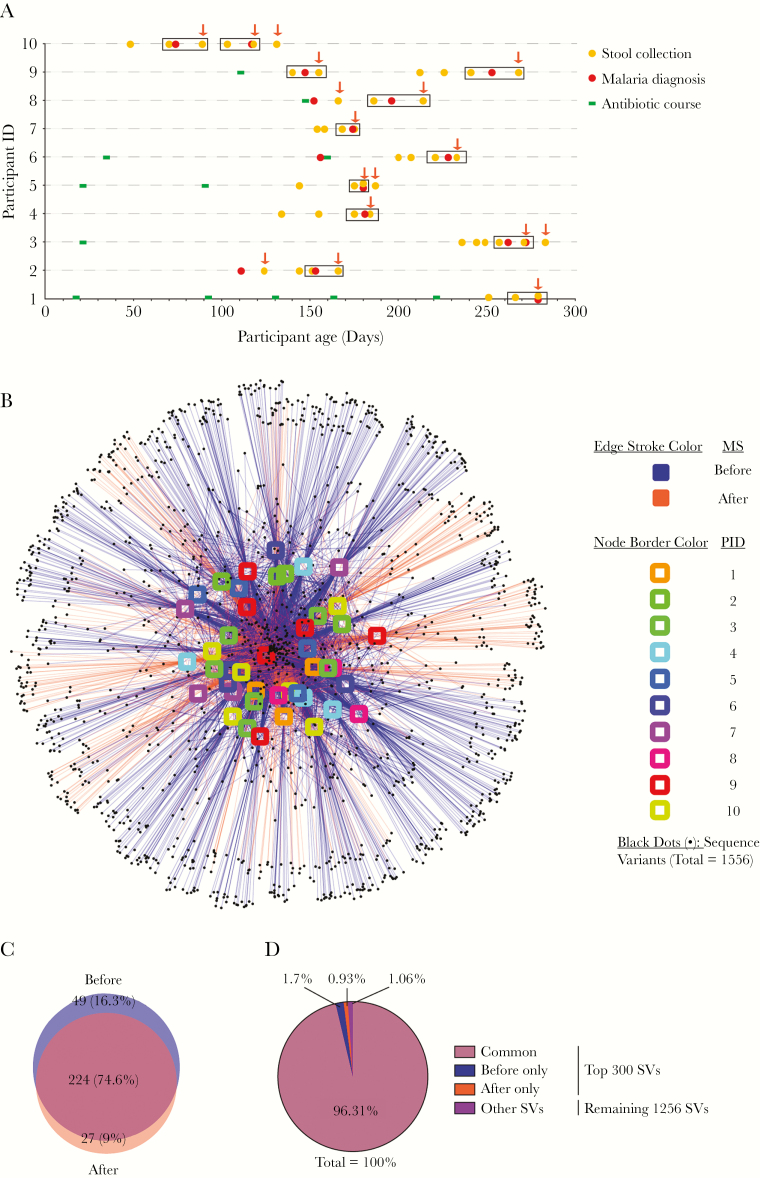
Observed taxonomic unit interaction maps show no distinct pattern between before and after malaria episode/artemether-lumefantrine (AL) treatment stool samples. (A) Timing of selected stool samples, malaria episodes, and antibiotic courses. Dates of stool collection (n = 44), malaria episodes/AL treatment (n = 16) and antibiotic courses (n = 12) are identified for each participant. Stool samples inside rectangles were selected for paired analysis between a single before (within 14 days) and after (within 18 days) malaria episode/AL treatment. Arrows mark all samples grouped as after (within 18 days) malaria/AL samples. All the events that took place after the last stool collection from each participant were censored and are therefore not depicted here. Of note, for each participant, no malaria episode occurred within 18 days of the final selected stool sample. (B) Interaction between 44 samples and 1556 sequence variants (SVs). Nodes (SVs) shared by the most samples are placed at the core of map as indicated by the number of edges connected to the nodes. (C) Overall top 300 SVs shared between the before and after malaria episode/AL treatment stool samples in Kenyan infants, which represents the core of interaction map. (D) Relative abundance of sequence variants shown in B and the remaining 1256 SVs.

A total of 1234 stool samples were collected during the cohort. Forty-four of these—from 10 participants—met the selection criteria for this study (Supplementary Table 1; Figure 1A). Six of 10 participants had 1 or more antibiotic courses before the first selected stool sample (Figure 1A). All antibiotic courses lasted 5 days (Supplementary Table 2). All 16 malaria episodes were nonsevere (ie, not requiring admission to hospital) and treated with a 3-day course of AL starting on the day of diagnosis. Rapid diagnostic test was positive for all 16 episodes. *Plasmodium falciparum* was detected by slide microscopy for 11 episodes, and no parasites were seen for 5 episodes (Supplementary Table 3). In some analyses, paired stool samples from a single before (within 14 days) and after (within 18 days) malaria episode were compared (Figure 1A, boxed samples). This paired analysis subset comprised 24 stool samples.

### Gut Bacterial Communities Are Not Affected by Acute Febrile Malaria Artemether-Lumefantrine Treatment

Demultiplexing the raw sequencing reads from an amplicon spanning V3–V4 of the 16S rRNA gene in all 44 stool samples (Figure 1A) produced an average of 248 577.8 (standard error ± 7531.72) and median of 251 803 reads per sample (minimum = 50 800 and maximum = 335 554) totaling 10 937 425 reads (Supplementary Table 1). Analysis of the reads identified that the forward reads outperformed the joined reads and were used for all the analysis performed in this study (Supplementary Text and Supplementary Figure 1).

Because every episode of malaria was treated with the standard 3-day AL course, malaria episodes and AL treatment were treated synonymously when comparing bacterial communities in stool samples collected before and after these reference time points. Observed taxonomic unit (OTU) interaction map showed no distinct pattern of shared sequence variants (SVs) between before and after malaria/AL episodes among all 44 stool samples (Figure 1B). Furthermore, the top 300 abundant SVs, which represents the core of OTU interaction map (98.92% of all SVs), had 224 (74.6%) SVs in common between the before and after malaria/AL (Figure 1C), representing 96.31% of total SVs detected in the infant stool samples (Figure 1D). Likewise, paired analysis of a single stool sample collected before and after a malaria/AL episode (12 pairs of samples between the 10 participants) (Figure 1A) showed no clear clustering of stool samples based on the shared SVs (Supplementary Figure 2A). The top 300 abundant core SVs (99.61% of all SVs) had 194 (64.7%) SVs in common between before and after malaria/AL stool samples (Supplementary Figure 2B). This constituted 95.8% of all SVs in the infant stool samples (Supplementary Figure 2C).

**Figure 2.**
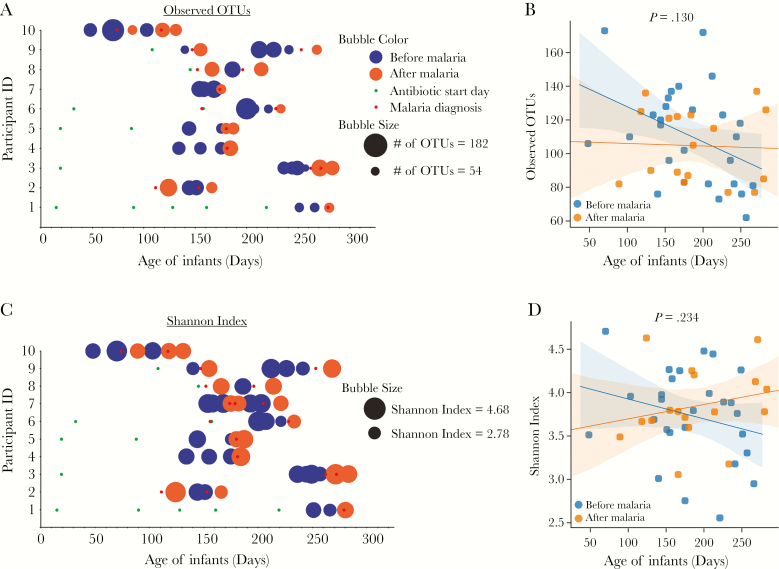
Alpha diversity analysis reveals no difference in bacterial communities in Kenyan infants before or after malaria episodes/artemether-lumefantrine (AL) treatment. Bubble plot (A) and dot plot (B) of Observed_OTUs. Bubble plot (C) and dot plot (D) of Shannon index. Statistical analysis was performed using linear mixed-effects model (LMEM). Shaded area represents 95% confidence interval.

Bacterial diversity, as measured by alpha diversity (within sample diversity) using Observed_OTUs (richness/number of species present) and Shannon index (richness and evenness), were analyzed between the before and after malaria/AL episodes. Neither Observed_OTUs (*P* = .130, linear mixed- effects model [LMEM]; Figure 2A and B) nor Shannon index (*P* = .234, LMEM; Figure 2C and D) were significantly different between the before and after malaria/AL samples. Furthermore, sex, antibiotic use, number of malaria episodes, antibiotic start day, and age had no effect on alpha diversity (measured using Observed_OTUs, pielou_e, and Shannon index) as shown in Supplementary Table 4. Discordant with previous studies showing an increase in bacterial alpha diversity with increasing age up to 2 years of age [16], in this study of only 10 infants, there was marginal negative correlation between infant age and Observed_OTUs (R^2^ = 0.09, *P* = .044; Supplementary Figure 3A) but no correlation between age and Shannon index (R^2^ = 0.007, *P* = .6) (Supplementary Figure 3B). Similar results were also observed when samples were segregated into those infants who did or did not have antibiotics through the last stool sample selected for this study (Supplementary Figure 3C and D).

**Figure 3.**
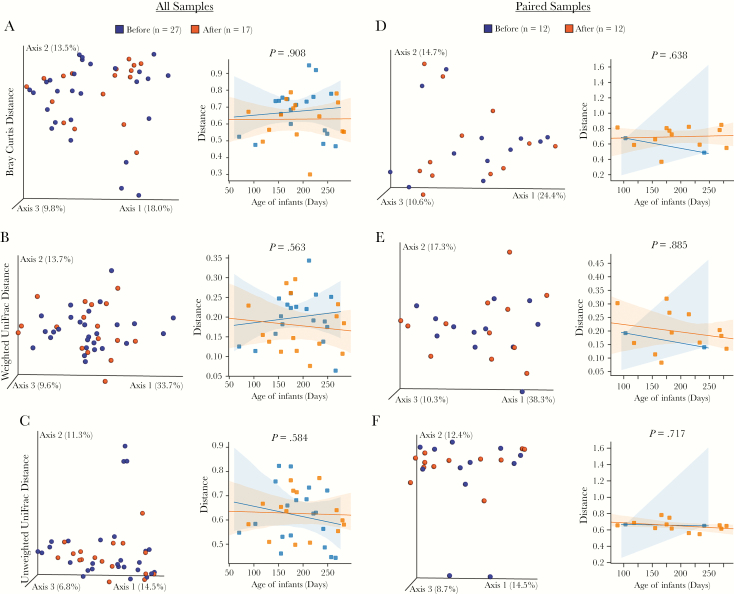
Beta diversity analysis reveals no difference in bacterial communities between Kenyan infants before or after malaria episodes/artemether-lumefantrine (AL) treatment. Beta diversity of all 44 samples (A–C) or 24 paired samples (D–F) measured by Bray-Curtis (A and D), weighted UniFrac distance (B and E), and unweighted UniFrac distance (C and F). Samples having similar gut microbiota composition are clustered together in the principal coordinates analysis plots. Statistical analysis was performed using linear mixed-effects model (LMEM).

Beta diversity (bacterial diversity between samples) was calculated using (1) non-phylogenetic Bray-Curtis distance and (2) phylogenetic weighted and unweighted UniFrac distance for all samples (n = 44, left column) and paired samples (n = 24, right column) (Figure 3). The bacterial community structure and composition between the before and after malaria/AL stool samples were not different in any of the samples or paired samples analysis using both non-phylogenetic and phylogenetic beta diversity metrics (*P* > .05, LMEM) (Figure 3). These analyses indicate that an acute malaria/AL episode had no impact on the structural composition of the stool bacteria community.

### Minimal Differentially Abundant Bacterial Features in Stool of Infants Before and After Malaria Artemether-Lumefantrine Episode

**Figure 4.**
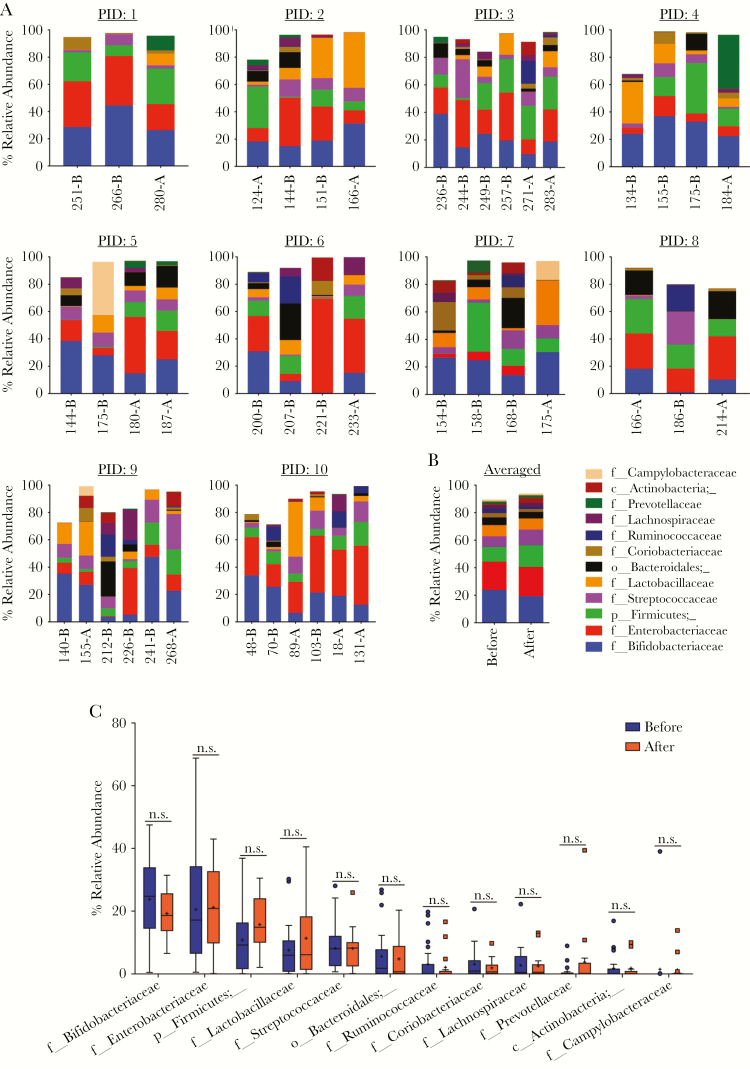
Taxonomic assignment at family level reveals no difference in bacterial communities in the before and after malaria episode/artemether-lumefantrine (AL) treatment samples. (A) Relative abundance of top 12 bacterial families in each stool sample from each participant. x-axis: arranged according to the age (days) of participant at time of stool collection. (B) Averaged relative abundance of top 12 bacterial families. (C) Relative abundance of each of the top 12 bacterial families compared between before and after malaria episode/AL treatment stool samples. Boxes show the 25th and 75th percentile, line equals median, asterisk equals mean, and point outside the whisker are outliers. Data were analyzed by unpaired *t* test. NOTE: Not all of the sequence reads were not classified up to the family level. A, after malaria; B, before malaria, PID, participant identification.

The prior analyses did not identify major changes in the bacterial community structures in stool samples collected before and after malaria/AL episodes. Yet, smaller taxonomic differences may have been present between the stool samples. Graphical phylogenetic analysis showed similar taxonomic profile between before and after malaria/AL (Supplementary Figure 4). In addition, linear discriminate effect size (LEfSe) analysis was used to identify any differentially abundant bacteria at the phylum, class, order, family, genus, and SV level between the before and after malaria/AL stool samples. The LEfSe analysis identified discriminative bacterial features with a 2 threshold cut off on the logarithmic scale (*P* < .05, Wilcoxon and Kruskal-Wallis test) (Supplementary Figure 5). However, when accounted for repeated measures, *Fusobacteriaceae* was the only feature that was different (higher after malaria/AL treatment; *P* = .025, LMEM). Consistently, analysis of the relative abundance of the top 12 bacteria family among the individual participants revealed no gross pattern between the before and after malaria/AL stool samples (Figure 4A). Similar results were seen when the overall abundance of these 12 bacterial families were analyzed between the combined before and after malaria/AL stool samples (Figure 4B). When each of the top 12 bacteria families were individually compared between the before and after malaria/AL stool samples, none of them were significantly different (*P* > .05, unpaired *t* test) (Figure 4C). Likewise, the heat map and principal coordinates analysis (PCoA) plot (Supplementary Figure 6A and B) of the top 16 genera did not show clustering of the before and after malaria/AL stool samples.

**Figure 5.**
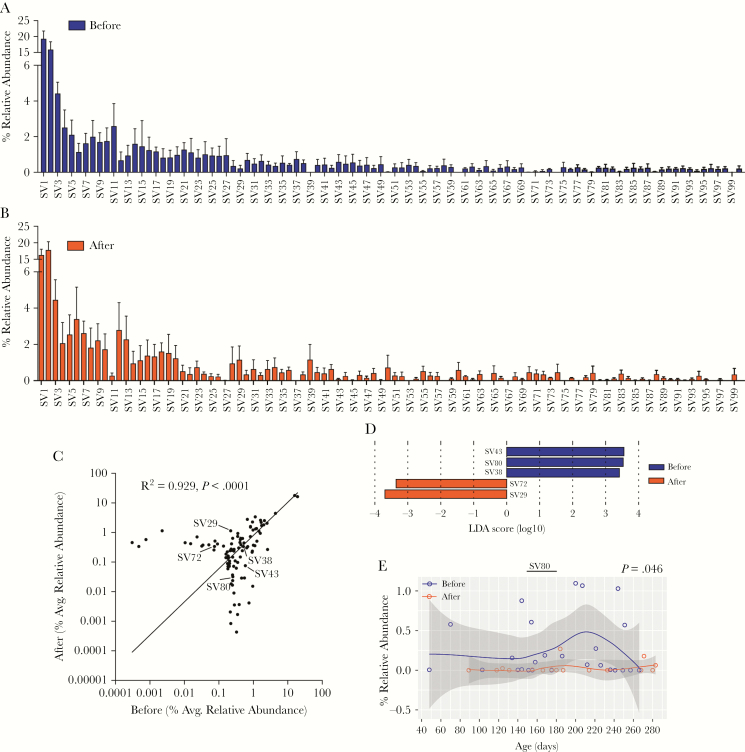
Taxonomic assignment at sequence variant (SV) level reveals minimal differences between the before and after malaria episode/artemether-lumefantrine (AL) treatment stool samples. (A and B) Relative abundance of overall top 100 SVs. (C) Pearson correlation of those 100 SVs. Sequence variants marked with an arrow were significantly differentially abundant before and after malaria episode/AL treatment as shown by linear discriminate effect size analysis (D). Relative abundance of SV80 and significance level tested using linear mixed-effects model.

Analysis at the SV level identified minimal differences between the before and after malaria/AL stool samples. A majority of the top 100 SVs, which represents 91.45% and 90.98% of total SVs in the before and after samples, respectively, had a similar relative abundance between the before and after malaria/AL stool samples (Figure 5A and B), which correlated (R^2^ = 0.948, *P* < .0001) (Figure 5C). The LEfSe analysis identified only 5 differentially abundant SVs (Figure 5D). SV43, SV80, and SV38 were abundant in stool samples before malaria/AL, whereas SV29 and SV72 were abundant in stool samples after malaria/AL (Figure 5D). However, only SV80 was different when the repeated measures were taken into consideration (*P* = .046, LMEM) (Figure 5E, Supplementary Figure 7). The top BLASTN (v2.7.1+) hit against the nucleotide database for SV43, SV80, SV38, SV72, and SV29 were *E coli* strain Ecol_746, *Erysipelatoclostridium ramosum* strain CCBE 141-17, *Streptococcus pneumoniae* ATCC 49619, *Lactobacillus fermentum* strain S1B1.25, and *Lachnospiraceae bacterium*, respectively. These analyses demonstrate that malaria/AL episodes have limited effects on stool bacteria populations at the SV level.

### Malaria Artemether-Lumefantrine Have Minimal Impact on Predicted Functional Activity of Stool Bacterial Communities

**Figure 6.**
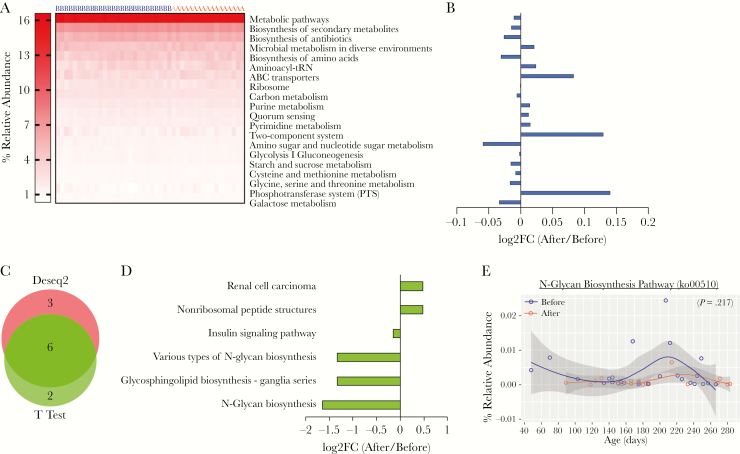
Predicted metagenomic capacity at KEGG pathway level reveals no significant different pathway. Metagenomic capacity was predicted using Piphillin. (A) Heat map depicts the relative abundance of top 20 KEGG pathway before and after malaria episode/artemether-lumefantrine (AL) treatment and (B) the log_2_-fold change (log2FC) of these pathways. (C) KEGG pathways were normalized with DeSeq2, and differentially abundant pathways were analyzed with DeSeq2 and *t* test (*P* < .05) and (D) fold change (FC) of pathways common to both methods. (E) Relative abundance of *N*-glycan biosynthesis pathway having highest fold change. Data were analyzed by linear mixed-effects model. A, after malaria sample; B, before malaria sample.

The metagenome in the stool samples was predicted using the online tool Piphillin. None of the top 20 most highly abundant KEGG pathways (Figure 6A) were more abundant than ± 0.13 (log2FC) (Figure 6B). KEGG pathways were normalized with DeSeq2, and differentially abundant pathways were then calculated using both DeSeq2 and *t* test with the web portal, RNA-Seq 2G (http://rnaseq2g.awsomics.org). Nine and eight pathways were enriched in stools before and after malaria/AL using DeSeq2 and *t* test, respectively (*P* < .05) (Figure 6C). Six pathways were common to both tests, which was enriched in stool samples before (4 pathways) and after (2 pathways) malaria/AL (Figure 6D). Only *N*-glycan biosynthesis had log2FC <1.5 before and after malaria/AL stool samples (Figure 6D), but this difference was lost when the percentage relative abundance was compared using LMEM (*P* = .217) (Figure 6E).

In addition, the metagenomic capacity of infants with malaria at the KEGG orthology (KO gene) level was analyzed. The inferred metagenome at gene level were normalized with DeSeq2, and differentially abundant genes were calculated using DeSeq2 and *t* test using RNA-Seq 2G, resulting in 47 genes common to both tests (Supplementary Figure 8A–C). All were abundant in before malaria/AL samples. Stool samples were clustered using these differentially abundant genes, yet PCoA and heat map analysis showed overlapping clustering of the stool samples, with the exception of 4 before malaria/AL stool samples (Supplementary Figure 8D–E). Among these 47, most did not fall into any KO pathway (Supplementary Figure 8F). Inconsistent with the KEGG pathway analysis (Figure 6), the pathway with the most gene hits was not *N*-glycan biosynthesis (Supplementary Figure 8F). Moreover, after visualization of the *N*-glycan biosynthesis pathway with Pathview (https://pathview.uncc.edu/), only 3 KO were inferred by Piphillin of 43 KO in the pathway (Supplementary Figure S9). However, many KOs of histidine metabolism pathway were enriched (Supplementary Figure S8 and S9). Collectively, these data provide no compelling evidence that malaria/AL episodes had any effect on the potential functional activity of stool bacteria communities.

### Number of Malaria Episodes Was the Only Significant Covariate Within the Stool Sample Bacteria Populations

**Figure 7.**
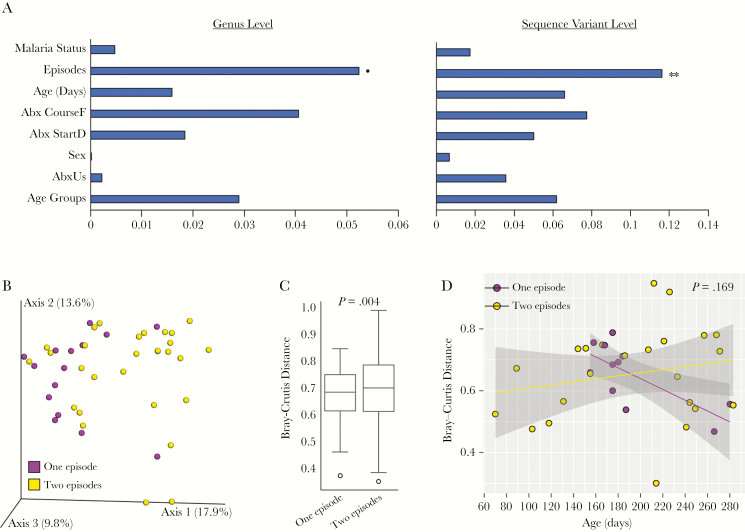
Number of malaria episodes loosely explain variation in stool samples. (A) Variance and significance explained by covariates modeled using EnvFit at genus level and sequence variant level using Bary-Curtis distance. Horizontal bars show variance (r2) explained by covariates (see Supplementary Table S1 for subgroupings). (B) Principal coordinates analysis plot of Bray-Curtis distance of stool samples between children with 1 versus 2 malaria episodes. Box plot (C) and scatter plot (D) show significance level using permutation-based multivariate analysis of variance and linear mixed-effects model, respectively. Shaded area of scatter plot represents 95% confidence interval. **P = .005; *P = .095.

Finally, variance (r^2^) explained by various covariates in this study was calculated using EnvFit implemented in vegan R package. Earlier, we showed no difference in alpha diversity among groups of various covariates (Supplementary Table 4). Interestingly, using the Bray-Curtis distance matrix at the genus and SV levels, only the number of malaria episodes explained statistically significant variation in the bacteria composition of stool samples (Figure 7A). Furthermore, the PCoA plot showed distinct clustering of stool samples between infants with 1 malaria episode compared with 2 malaria episodes (*P* = .004, permutation-based multivariate analysis of variance) (Figure 7B and C). However, the significance was lost between 1 versus 2 malaria episodes when accounting for the repeated measure (*P* = .169, LMEM) (Figure 7D).

## DISCUSSION

This report provides the first longitudinal analysis of stool bacteria communities before and after a clinical malaria episode plus AL treatment in humans. The data identified minimal changes in gut microbiota of Kenyan infants due to malaria/AL. In contrast to these data in humans, 2 previous studies in rodent malaria models have reported larger shifts in the gut microbiota of mice before and after malaria infection associated with intestinal inflammation. Infection of mice (C57BL/6) with rodent *Plasmodium* species (*Plasmodium berghei* ANKA and *P yoelii nigeriensis*) induced pathological changes in the intestine including infiltration of inflammatory macrophages, T cells, detachment of epithelia in the small intestine, increased expression of inflammatory cytokines, and intestinal permeability [5, 6]. By contrast, no such intestinal inflammation and shift in gut microbiota was observed in *P berghei* ANKA-infected BALB/c mice [5], and *P yoelii nigeriensis* infection in CBA mice induces anti-inflammatory interleukin (IL)-10 [6, 17]. Likewise, in a nonhuman primate model—rhesus macaques (*Macaca mulatta*) infected with *Plasmodium fragile*—the parasite suppressed gut inflammation via the induction of IL-10, which blunted the influx of neutrophils in the gut [17]. Finally, individuals with severe pediatric malaria lack symptoms of gastroenteritis [17, 18]. Taken together, these data indicate that inflammation, intestinal damage, and changes in gut microbiota might be attributed to complex interactions between host genotype and *Plasmodium* species.

Adults with *P falciparum* infection have been shown to have increased gastrointestinal permeability lasting for a couple of days depending on the severity of malaria and treatment, which reverts to normal during convalescence [19]. In spite of the relative lack of clinical features of gastroenteritis, malaria is a major risk factor for invasive nontyphoid salmonellosis in sub-Saharan Africa [20]. Various mechanisms have been proposed that explain why the host immune system struggles to eliminate systemic *Salmonella* during malaria, including macrophage dysfunction due to ingestion of the malaria pigment hemozoin [21], impaired neutrophil oxidative burst [22], dysfunctional spleen in young children, and competition between bacteria and damaged red blood cells for phagocytic cells [23] among others [24]. However, it is less clear how *Salmonella* initially breaches the intestinal barrier to cause a systemic infection during malaria. It is well established that dysbiosis of the normal gut flora provides an opportunity for *Salmonella* to establish infection [8], and it might contribute to nontyphoid salmonellosis during malaria. Consistent with this possibility, *P yoelii nigeriensis*-induced dysbiosis resulted in increased susceptibility to *Salmonella typhimurium enterica* infection in C57BL/6 mice [6]. In contrast, the analyses reported here identified little to no change in stool bacteria in Kenyan infants after nonsevere malaria episodes. In addition to dysbiosis, *P yoelii nigeriensis* infections induce elevated production of intestinal IL-10, which facilitates increased translocation of *S typhimurium enterica* out of the intestinal tract [17]. Taken together, these results suggest that in humans *Plasmodium*-induced changes in the anti-inflammatory status of the intestinal tract may be more likely to contribute to intestinal translocation of *Salmonella* rather than gut microbiota dysbiosis.

Of the 16 malaria episodes, all were RDT positive but only 11 were slide positive. Copresence of fever and slide positivity amongst infants in this moderate to high transmission intensity setting is strongly indicative of acute clinical malaria rather than incidental parasitaemia [25]. Suspicion is therefore high that these 11 fever episodes were indeed acute *P falciparum* infection. Where slide is negative but RDT positive, suspicion may be lower. The RDT used detects the *P falciparum*-specific surface antigen histidine-rich protein 2 (HRP-2) in blood. The HRP2-based RDTs can remain positive for up to 42 days or beyond after the beginning of a treated clinical malaria episode [26]. Participants 1 and 4 each had only 1 malaria diagnosis throughout follow-up. Participant 1 had a negative RDT 6 days before their index malaria diagnosis date. For participant 4, RDTs done 16 and 48 days after the index diagnosis date were positive and then negative, respectively. Participant 8’s second positive RDT occurred 44 days after their last positive slide. These 3 circumstances are therefore in keeping with the index positive RDT likely indicating the start of an episode of clinical malaria despite slide negativity. The same could perhaps not be said for the second episodes for participants 3 and 10. These positive RDTs (negative slides) occurred 10 and 13 days, respectively, after a positive slide. However, these 2 episodes remain in the analysis because they conform to the prospectively determined malaria diagnostic criteria that follow World Health Organization guidelines [27]. For participant 3, the stool sample collected immediately before the second malaria episode is in fact classed as after the first episode (Figure 1A). Therefore, interpretation of this second slide-negative fever episode has no bearing on classification of stool samples. Likelihood of missed microscopy positive parasitaemia was hopefully very low in this study given the diligent passive and active case detection, with fieldworkers and phlebotomists continuously resident and available in participants’ villages.

Prior studies have identified a positive correlation between microbial diversity, measured by alpha diversity, and age of infants [16]. In contrast, our analysis did not identify a robust significant correlation in these participants (Supplementary Figure S3). One explanation may be that—as is frequently observed in this setting—many infants in this study had received antibiotics (even if in this study, by merit of the stool selection criteria, all of these courses occurred prior to the “before” stool samples). Another possible explanation is that an association between diversity and age does exist, but it is confounded by other factors that are themselves correlated with both age and microbial diversity. Examples of confounding factors include acute illnesses such as diarrhea, subclinical enteropathogen carriage, diet, life style, antibiotic usage of infant’s mother during and even after pregnancy that can influence infant gut microbiota via mother’s breastmilk. Another example is nutritional status. Severe and even moderate acute malnutrition (low weight-for-height) has been shown amongst Bangladeshi children to be associated with failure of gut microbiota to diversify with age [28]. During monthly measurement of weight and length, 5 of the 10 participants fulfilled criteria for moderate or severe wasting (weight for length Z score ≤2) at 1 or more time points, and 4 fulfilled criteria for moderate or severe stunting (length for age Z score ≤2) (data not shown). It is not yet known whether stunting is associated with gut microbiota immaturity. A weaker association between age and diversity—as perhaps expected in this undernourished population—may be more difficult to detect with this relatively small sample size.

Only the number of malaria episodes explained significant variation in the gut microbiome composition of Kenyan infants. It is important to note that this observation has been made in a small sample size, and that significance is lost when repetitive sampling is taken into consideration. Nevertheless, it raises the exciting possibility that differences in gut bacteria may contribute to differential outcomes of malaria in children. Clearly, this possibility will need to be examined in the context of a larger and more definitive study that determines the ability of gut microbiota to modulate the severity of malaria in African children.

## CONCLUSIONS

We demonstrate for the first time that human malaria episodes and AL treatment result in a minimal shift in the gut microbiota of Kenyan infants, which is in contrast to what was observed in some murine models of malaria. These results suggest that changes in the inflammatory nature of intestinal tissue, in contrast to gut microbiota dysbiosis, during malaria may contribute to translocation of enteric bacteria and progression to bacteremia. This study also leaves open exciting, yet unanswered, questions regarding interactions between human gut microbiota and malaria. For example, are there different gut microbiota between healthy individuals and patients with severe malaria? Given the increased appreciation of the gut-brain axis, is there an interplay between gut microbiota and cerebral malaria? Can machine learning algorithms predict the gut microbiota composition associated with children prone to *Plasmodium* infections? Addressing these questions could make substantial improvements in developing novel approaches to prevent malaria-related fatalities.

## Supplementary Data

Supplementary materials are available at *The Journal of Infectious Diseases* online. Consisting of data provided by the authors to benefit the reader, the posted materials are not copyedited and are the sole responsibility of the authors, so questions or comments should be addressed to the corresponding author.

jiy740_suppl_Supplementary_Table_S1

jiy740_suppl_Supplementary_Table_S2

jiy740_suppl_Supplementary_Table_S3

jiy740_suppl_Supplementary_Table_S4

jiy740_suppl_Supplementary_Figure_S1

jiy740_suppl_Supplementary_Figure_S2

jiy740_suppl_Supplementary_Figure_S3

jiy740_suppl_Supplementary_Figure_S4

jiy740_suppl_Supplementary_Figure_S5

jiy740_suppl_Supplementary_Figure_S6

jiy740_suppl_Supplementary_Figure_S7

jiy740_suppl_Supplementary_Figure_S8

jiy740_suppl_Supplementary_Figure_S9

jiy740_suppl_Supplementary_Material

## Notes

***Acknowledgments.* **This manuscript is published with permission from the Director of the Kenya Medical Research Institute. We thank Dr. Yousef Abu-Kwaik for critical review of the manuscripts.

***Author contributions.* **R. K. M., R. J. C., J. A. B., F. M. N., and N. W. S. contributed to study concept and design. R. J. C. and J. W. collected stool samples. R. J. C., W. G., and J. M. N. carried out deoxyribonucleic acid extraction and sequencing. R. K. M. and N. W. S. contributed to sequence analysis and interpretation. R. K. M. and N. W. S. drafted the manuscript. R. K. M., R. J. C., J. A. B., F. M. N., W. G., and N. W. S. reviewed and edited the manuscript.

***Financial support.* **Sample collection was funded by the Wellcome Trust (Grant no. 103376/Z/13/Z; to R. J. C.). Laboratory work, data analysis and write-up were supported by funds from the University of Louisville and the National Institutes of Health (Grant no. 1R01AI123486; to N.W.S.).

***Potential conflicts of interest.* **All authors: No reported conflicts of interest. All authors have submitted the ICMJE Form for Disclosure of Potential Conflicts of Interest.
