# Supplementary material for: Longitudinal Analysis of Infant Stool Bacteria Communities Before and After Acute Febrile Malaria and Artemether-Lumefantrine Treatment
Source: J Infect Dis. 2018 Dec 24;220(4):687–98. doi: 10.1093/infdis/jiy740 (PMC6639600; doi:10.1093/infdis/jiy740)
Supplement: jiy740_suppl_Supplementary_Table_S1 [file jiy740_suppl_supplementary_table_s1.pdf]

Table S1. Metadata file with read numbers and alpha diversity indices

PID: Participant ID
MS: Malaria Status (B-Before malaria, A-After malaria)
Episodes: number of malaria episodes
DaysStool/Age: Day at when stool was collected / age of infants
Age: Age of infants were categorized into three group (L100- less than 100 days, 100-200: between 100 and 200 days, G200- greater than 200 days)
AbxStartD: Age at which antibiotic was first prescribed (L50 and G50- Abx was prescribed less and greater than 50 days of age respectively, NoAbx: Abx was not prescribed)
PairAna: Samples that were included in paired analysis (Yes-included, No- Not included)
AbxUs: Abx usage (Abx- antibiotic was administered to child during the study; NoAbx- no antibiotic was administered to child during the study)
DemuxRead#: No of read that belongs to each stool sample after demultiplxing
QualityReads: Quality reads produced by DADA2 to construct the feature table (for Run 3)
OTUs: Observed taxonomic units
pielou_e: Alpha diversity index that measures evenness of sample
Shannon: Shannon index

#SampleID	PID	MalariaStatus	Episodes	DaysStool/Age	Age	AbxStartD	Sex	PairedAna	AbxUs	DemuxRead#	QualityReads	OTUs	pielou_e	Shannon
k-566	1	B	One	266	G200	L50	F	yes	Abx	234812	147,524	75	0.4689005	2.920696
k-617	1	A	One	280	G200	L50	F	yes	Abx	243206	160,474	77	0.602489	3.77567
k-481	1	B	One	251	G200	L50	F	No	Abx	268774	180,610	72	0.5698274	3.515792
k-91	2	A	Two	124	100-200	NoAbx	M	No	NoAbx	180706	115,354	134	0.6534227	4.617143
k-162	2	B	Two	144	100-200	NoAbx	M	No	NoAbx	201081	133,219	114	0.5786588	3.953912
k-276	2	A	Two	166	100-200	NoAbx	M	yes	NoAbx	221622	159,462	85	0.4739621	3.037809
k-193	2	B	Two	151	100-200	NoAbx	M	yes	NoAbx	264122	178,579	116	0.5263401	3.609631
k-655	3	B	Two	236	G200	L50	M	No	Abx	203493	129,364	97	0.5888144	3.886124
k-701	3	B	Two	244	G200	L50	M	No	Abx	227878	156,488	101	0.5607681	3.733713
k-850	3	A	Two	283	G200	L50	M	No	Abx	251972	167,557	110	0.5923192	4.016729
k-710	3	B	Two	249	G200	L50	M	No	Abx	267219	175,380	113	0.626757	4.274595
k-812	3	A	Two	271	G200	L50	M	yes	Abx	285600	179,420	128	0.6582701	4.607891
k-752	3	B	Two	257	G200	L50	M	yes	Abx	330868	226,347	54	0.5753316	3.310969
k-446	4	B	One	155	100-200	NoAbx	M	No	NoAbx	206094	131,028	95	0.5427935	3.566075
k-533	4	B	One	175	100-200	NoAbx	M	yes	NoAbx	211020	136,950	94	0.553001	3.624694
k-364	4	B	One	134	100-200	NoAbx	M	No	NoAbx	244433	160,738	103	0.5523107	3.693026
k-584	4	A	One	184	100-200	NoAbx	M	yes	NoAbx	291540	188,588	119	0.6188963	4.267177
k-563	5	A	One	180	100-200	L50	M	yes	Abx	231308	157,989	86	0.5622702	3.613297
k-590	5	A	One	187	100-200	L50	M	No	Abx	235981	160,500	93	0.6403142	4.187116
k-400	5	B	One	144	100-200	L50	M	No	Abx	248015	159,933	112	0.5848735	3.981442
k-543	5	B	One	175	100-200	L50	M	yes	Abx	335554	223,217	78	0.4427931	2.783133
k-895	6	A	Two	233	G200	L50	F	yes	Abx	251634	163,442	73	0.5135767	3.178949
k-862	6	B	Two	221	G200	L50	F	yes	Abx	253209	172,533	69	0.4170114	2.547324
k-804	6	B	Two	207	G200	L50	F	No	Abx	269899	168,396	80	0.6298644	3.981957
k-784	6	B	Two	200	100-200	L50	F	No	Abx	301243	197,601	164	0.6086661	4.478292
k-702	7	A	One	175	100-200	NoAbx	F	yes	NoAbx	180384	117,739	77	0.5939432	3.722115
k-613	7	B	One	158	100-200	NoAbx	F	No	NoAbx	241638	159,075	122	0.6024591	4.175486
k-673	7	B	One	168	100-200	NoAbx	F	yes	NoAbx	259770	168,769	133	0.5993051	4.228267
k-600	7	B	One	154	100-200	NoAbx	F	No	NoAbx	326196	191,034	124	0.6160619	4.284215
k-757	8	A	Two	166	100-200	G50	M	No	Abx	247014	164,180	115	0.5539469	3.792038
k-926	8	A	Two	214	G200	G50	M	yes	Abx	268933	176,346	109	0.5622512	3.80542
k-828	8	B	Two	186	100-200	G50	M	yes	Abx	282181	188,660	123	0.5444813	3.780069
k-1069	9	A	Two	268	G200	G50	F	yes	Abx	50800	33,645	76	0.65884	4.116385
k-1030	9	B	Two	241	G200	G50	F	yes	Abx	212677	147,329	72	0.5166588	3.187746
k-679	9	B	Two	140	100-200	G50	F	yes	Abx	261137	179,568	65	0.501034	3.017411
k-990	9	B	Two	226	G200	G50	F	No	Abx	279817	182,145	122	0.5613421	3.890515
k-744	9	A	Two	155	100-200	G50	F	yes	Abx	280671	166,444	104	0.5664421	3.795411
k-956	9	B	Two	212	G200	G50	F	No	Abx	290865	180,349	132	0.6266758	4.414551
k-692	10	A	Two	89	L100	NoAbx	M	yes	NoAbx	185188	115,858	74	0.5635802	3.499525
k-858	10	A	Two	131	100-200	NoAbx	M	No	NoAbx	185527	112,077	94	0.5623427	3.685925
k-505	10	B	Two	48	L100	NoAbx	M	No	NoAbx	251459	171,379	103	0.5279912	3.530414
k-609	10	B	Two	70	L100	NoAbx	M	yes	NoAbx	259379	170,192	170	0.632021	4.682891
k-816	10	A	Two	118	100-200	NoAbx	M	yes	NoAbx	302668	204,587	114	0.5331983	3.643285
k-762	10	B	Two	103	100-200	NoAbx	M	yes	NoAbx	309838	210,737	105	0.5900997	3.962075
