# Supplementary material for: Longitudinal Analysis of Infant Stool Bacteria Communities Before and After Acute Febrile Malaria and Artemether-Lumefantrine Treatment
Source: J Infect Dis. 2018 Dec 24;220(4):687–98. doi: 10.1093/infdis/jiy740 (PMC6639600; doi:10.1093/infdis/jiy740)
Supplement: jiy740_suppl_Supplementary_Table_S2 [file jiy740_suppl_supplementary_table_s2.pdf]

Table S2. Antibiotic course

participant ID	age at start date (days)	antibiotic name	prescribed duration (days)
1	15	amoxicillin	5
1	90	metronidazole	5
1	128	amoxicillin	5
1	161	cotrimoxazole	5
1	219	cloxacillin	5
3	19	cotrimoxazole	5
5	19	amoxicillin	5
5	88	cotrimoxazole	5
6	32	amoxicillin	5
6	157	cloxacillin	5
8	145	amoxicillin	5
9	108	amoxicillin	5

Note: Antibiotic course given after the last stool sample collected for this study is not shown.
