# Supplementary material for: Longitudinal Analysis of Infant Stool Bacteria Communities Before and After Acute Febrile Malaria and Artemether-Lumefantrine Treatment
Source: J Infect Dis. 2018 Dec 24;220(4):687–98. doi: 10.1093/infdis/jiy740 (PMC6639600; doi:10.1093/infdis/jiy740)
Supplement: jiy740_suppl_Supplementary_Table_S3 [file jiy740_suppl_supplementary_table_s3.pdf]

Supplemental Table 3: Malaria episode characteristics

PID: participant identification
RDT: rapid diagnostic test
WBCs: white blood cells
RBCs: red blood cells

PID	Date	Age (days)	Age (months)	RDT positive	P. falciparum per 200 WBCs	P. falciparum per 500 RBCs	P. malariae per 200 WBCs	P. ovale per 200 WBCs	gametes per 200 WBCs	passive or active case detection
1	6-Jun-16	279	9.147541	yes	0	0	0	0	0	active
2	21-Jan-16	111	3.639344	yes	23		0	0	0	active
2	3-Mar-16	153	5.016394	yes		30	0	0	0	active
3	11-Jul-16	262	8.590164	yes	319		0	0	0	active
3	21-Jul-16	272	8.918033	yes	0	0	0	0	0	active
4	24-May-16	181	5.934426	yes	0	0	0	0	0	active
5	24-May-16	180	5.901639	yes		6	0	0	0	passive
6	30-May-16	156	5.114754	yes		21	0	0	0	passive
6	10-Aug-16	228	7.47541	yes	337		0	0	0	passive
7	22-Jun-16	174	5.704918	yes	425		0	0	0	active
8	22-Jun-16	152	4.983606	yes	117		0	0	0	active
8	5-Aug-16	196	6.426229	yes	0	0	0	0	0	active
9	27-Jun-16	147	4.819672	yes	90		0	0	0	passive
9	11-Oct-16	253	8.295082	yes		19	0	0	0	passive
10	7-Jun-16	74	2.426229	yes		12	0	0	0	active
10	20-Jul-16	117	3.836066	yes	0	0	0	0	0	active
