# Supplementary material for: Longitudinal Analysis of Infant Stool Bacteria Communities Before and After Acute Febrile Malaria and Artemether-Lumefantrine Treatment
Source: J Infect Dis. 2018 Dec 24;220(4):687–98. doi: 10.1093/infdis/jiy740 (PMC6639600; doi:10.1093/infdis/jiy740)
Supplement: jiy740_suppl_Supplementary_Table_S4 [file jiy740_suppl_supplementary_table_s4.pdf]

Table S4: Alpha Diversity Analysis

<u>Abx Start Day</u>: The age at which first antibiotic course was started (L50 = Less than 50 days old; G50 = Greater than 50 days old; L100 = Less than 100 days old and 100-200 = Between 100 and 200 days old)

<u>Age</u>: The age of infants at stool collection were categorized into three groups (L100 = Less than 100 days old, 100-200 = Between 100 and 200 days old, and G200 = Greater than 200 days old)

			p-value		
			Richness	Evenness	Evenness + Abundance
Category	Group1	Group2	OTUs	pielou_e	Shannon
Malaria Status	Before(n=27)	After(n=17)	0.13	0.382	0.234
Sex	Male(n=27)	Female(n=17)	0.199	0.252	0.225
Abx Use	No(n=18)	Yes(n=26)	0.669	0.329	0.418
Malaria Episodes	1(n=15)	2(n=29)	0.273	0.428	0.312
Abx Start Day	L50(n=17)	G50(n=9)	0.906	0.425	NA
	G50(n=9)	NoAbx(n=18)	0.828	0.172	NA
Age Group	L100(n=3)	100-200 (n=24)	0.563	0.889	0.916
	G200(n=17)	100-200 (n=24)	0.915	0.605	0.721

Statistical analysis were performed using linear mixed effect models.
