# Supplementary figures and images for: Longitudinal Analysis of Infant Stool Bacteria Communities Before and After Acute Febrile Malaria and Artemether-Lumefantrine Treatment
Source: J Infect Dis. 2018 Dec 24;220(4):687–98. doi: 10.1093/infdis/jiy740 (PMC6639600; doi:10.1093/infdis/jiy740)

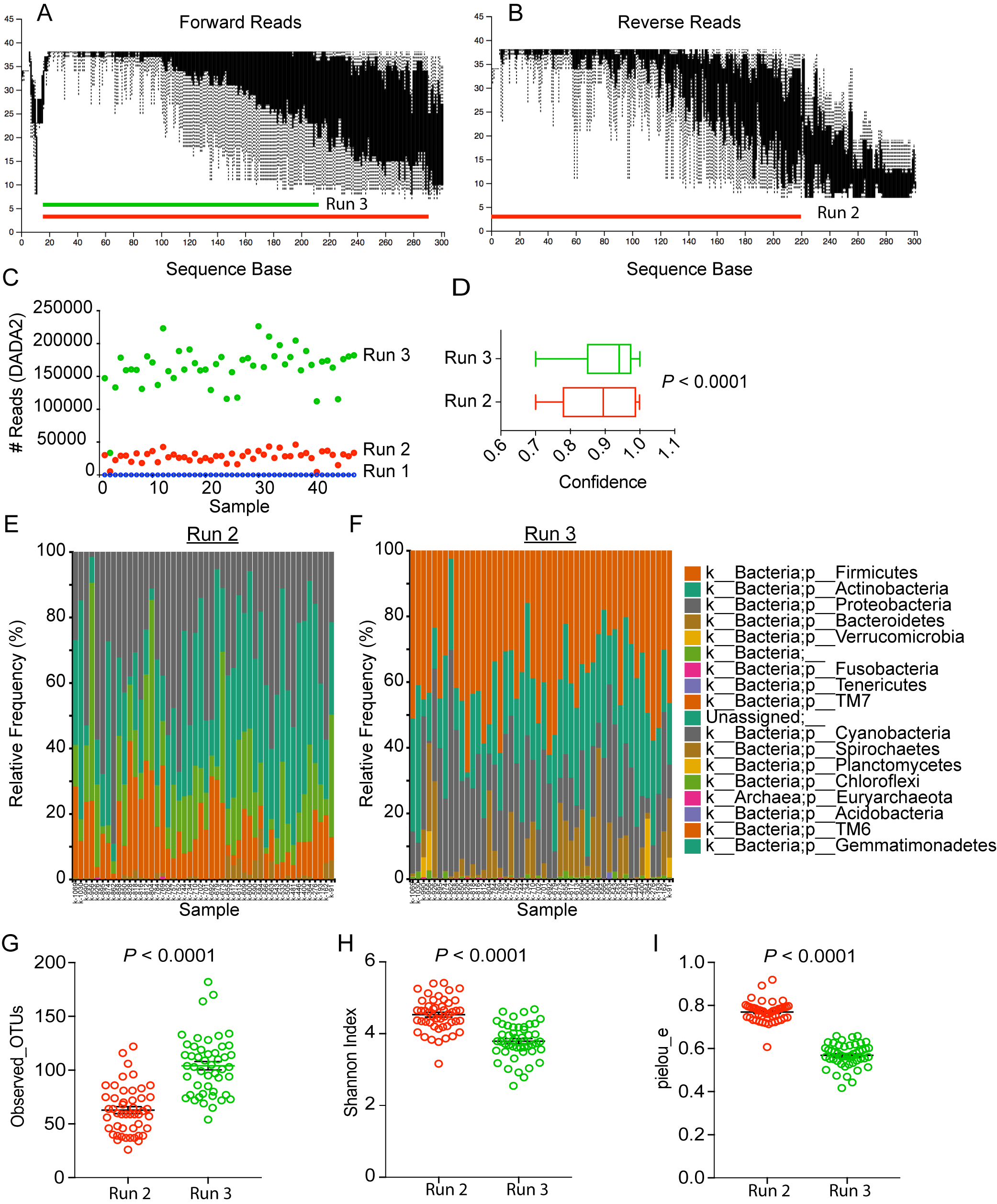

Supplement: jiy740_suppl_Supplementary_Figure_S1 [file jiy740_suppl_supplementary_figure_s1.png]

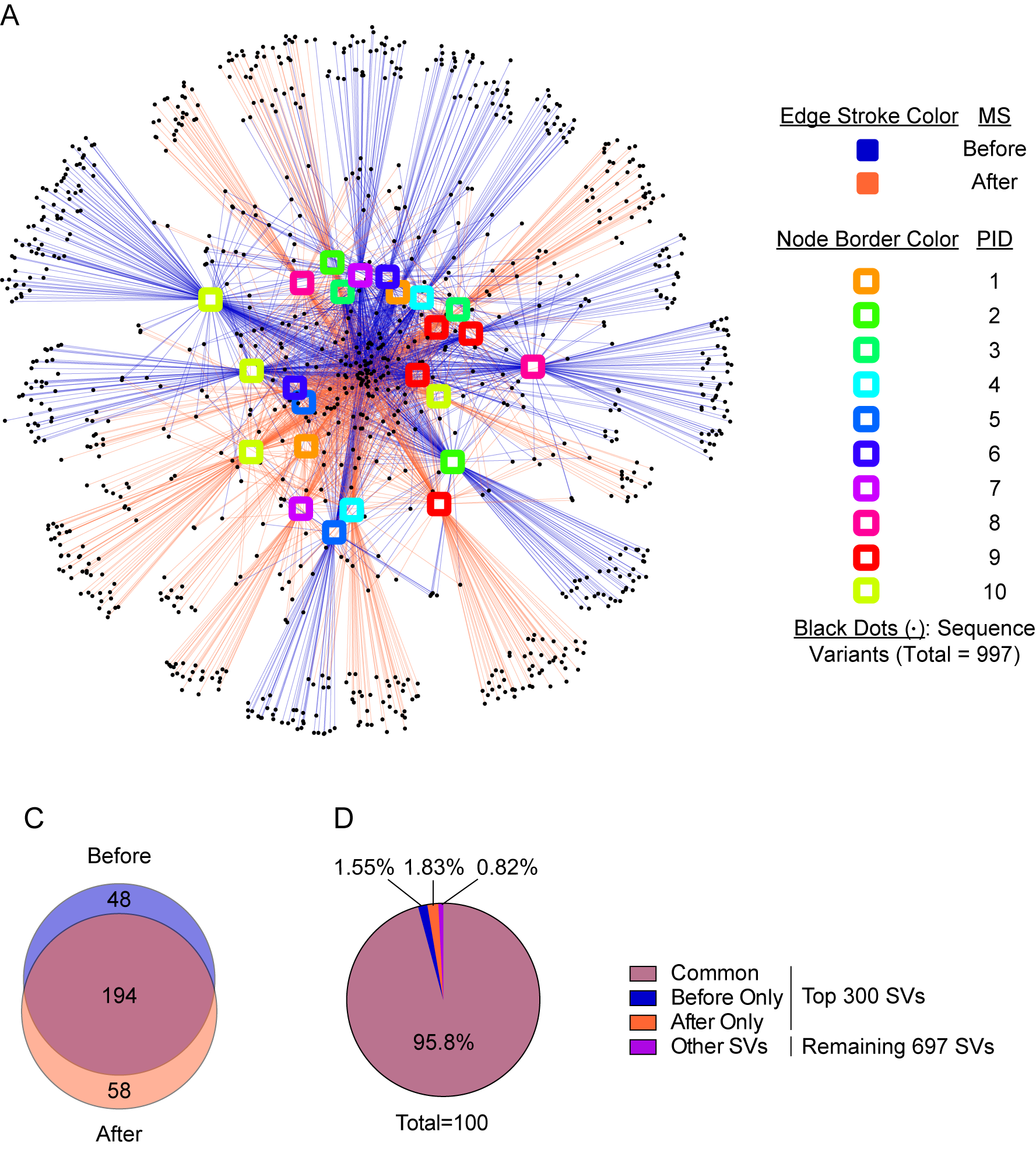

Supplement: jiy740_suppl_Supplementary_Figure_S2 [file jiy740_suppl_supplementary_figure_s2.png]

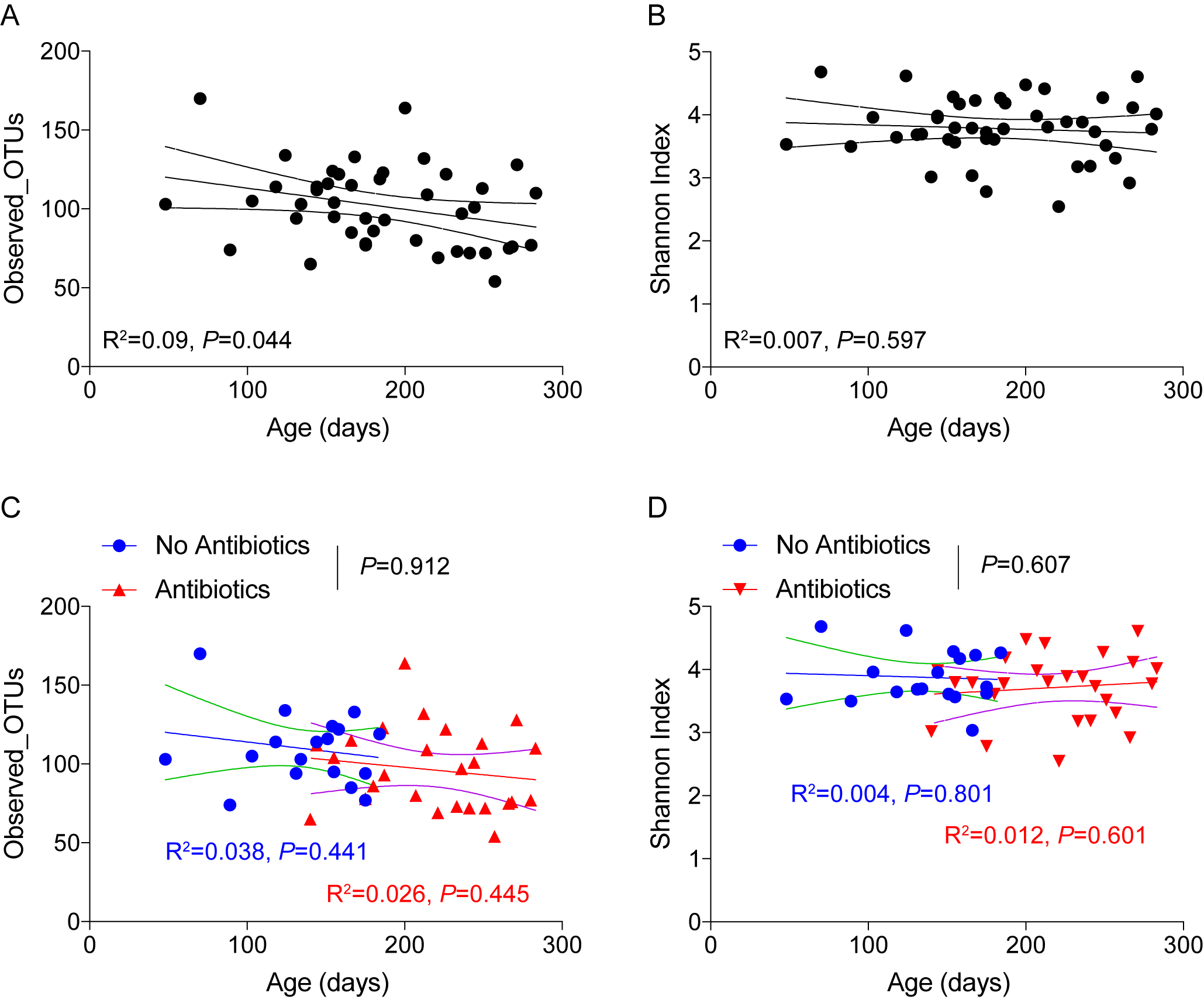

Supplement: jiy740_suppl_Supplementary_Figure_S3 [file jiy740_suppl_supplementary_figure_s3.png]

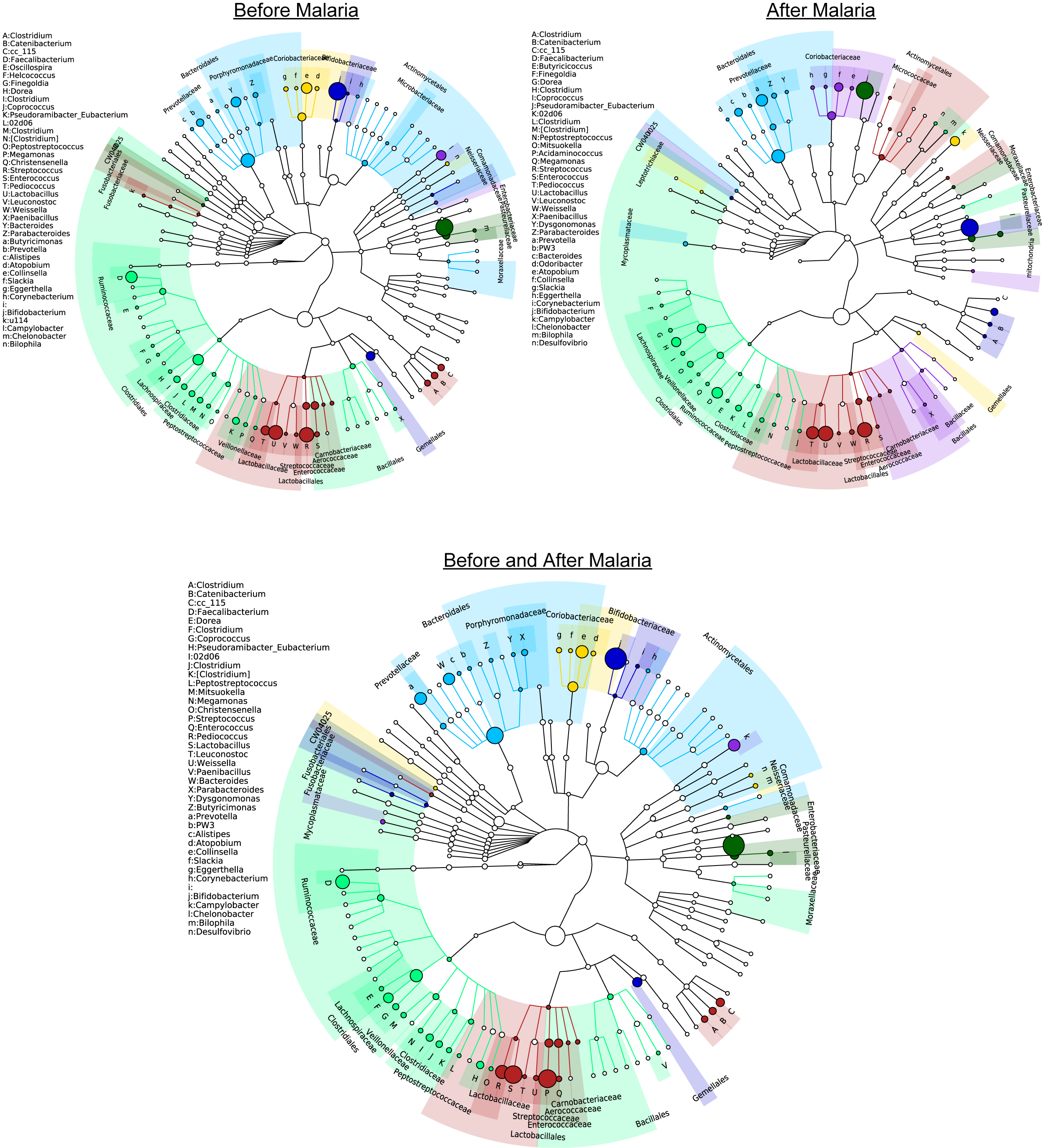

Supplement: jiy740_suppl_Supplementary_Figure_S4 [file jiy740_suppl_supplementary_figure_s4.png]

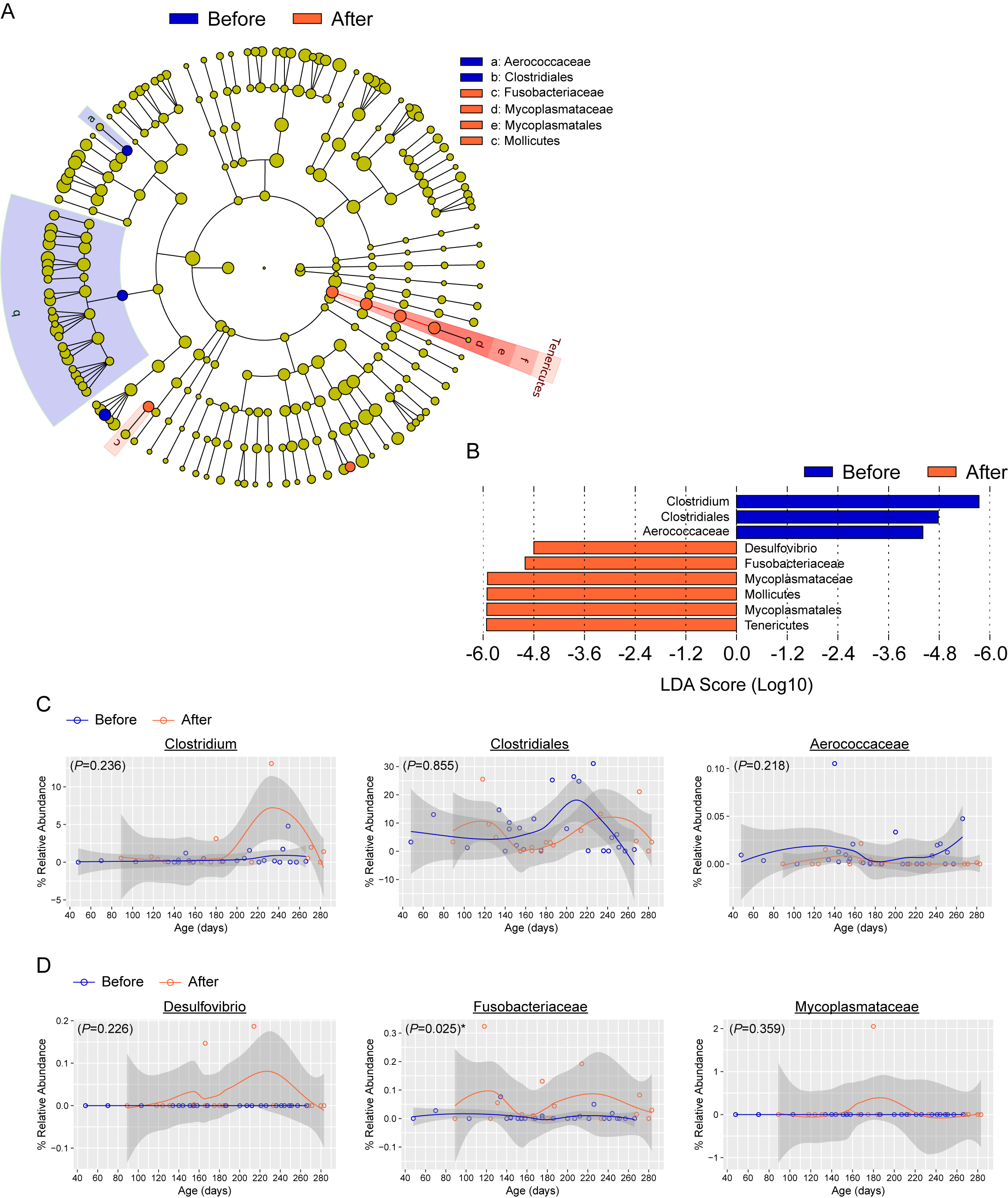

Supplement: jiy740_suppl_Supplementary_Figure_S5 [file jiy740_suppl_supplementary_figure_s5.png]

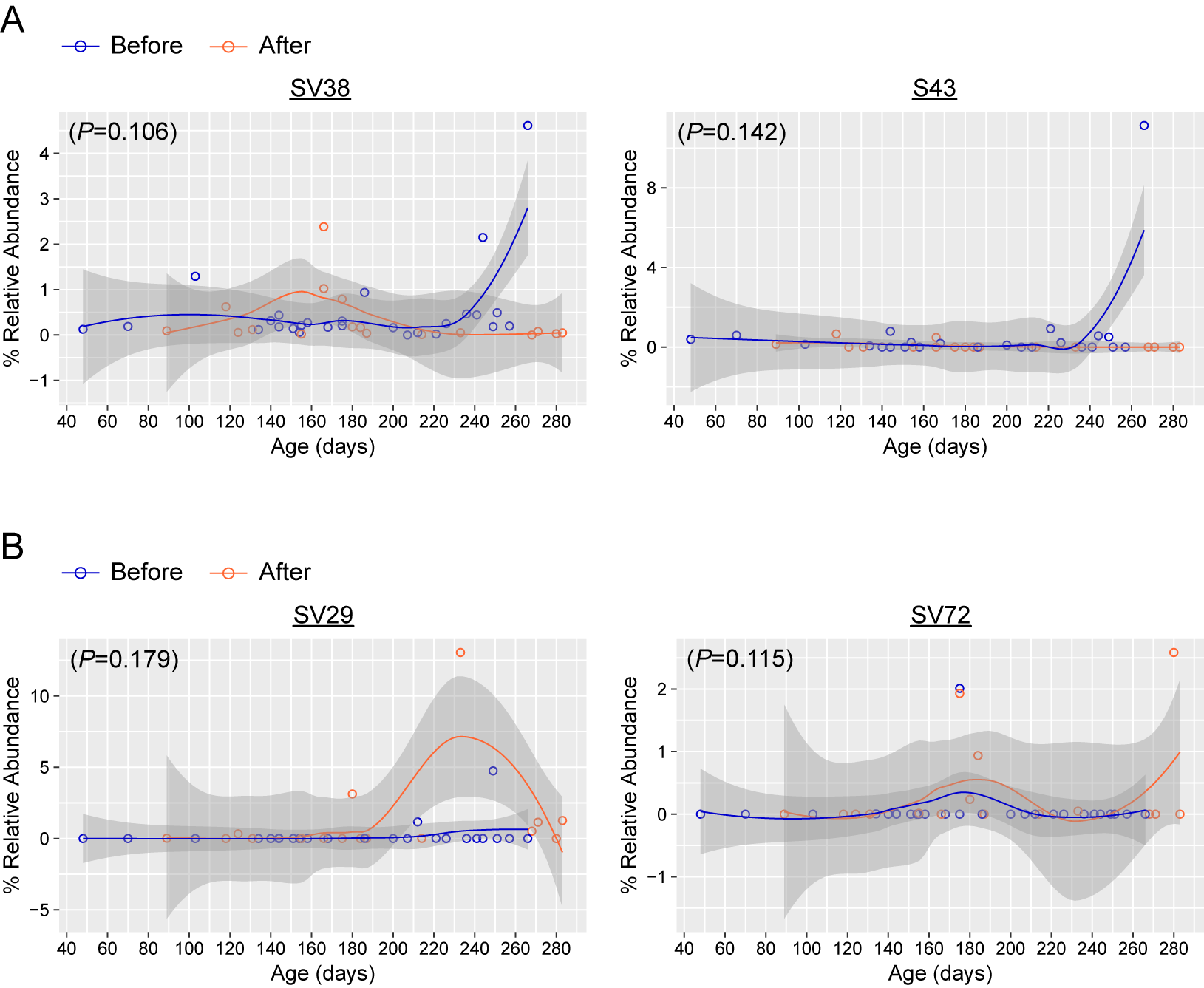

Supplement: jiy740_suppl_Supplementary_Figure_S6 [file jiy740_suppl_supplementary_figure_s6.png]

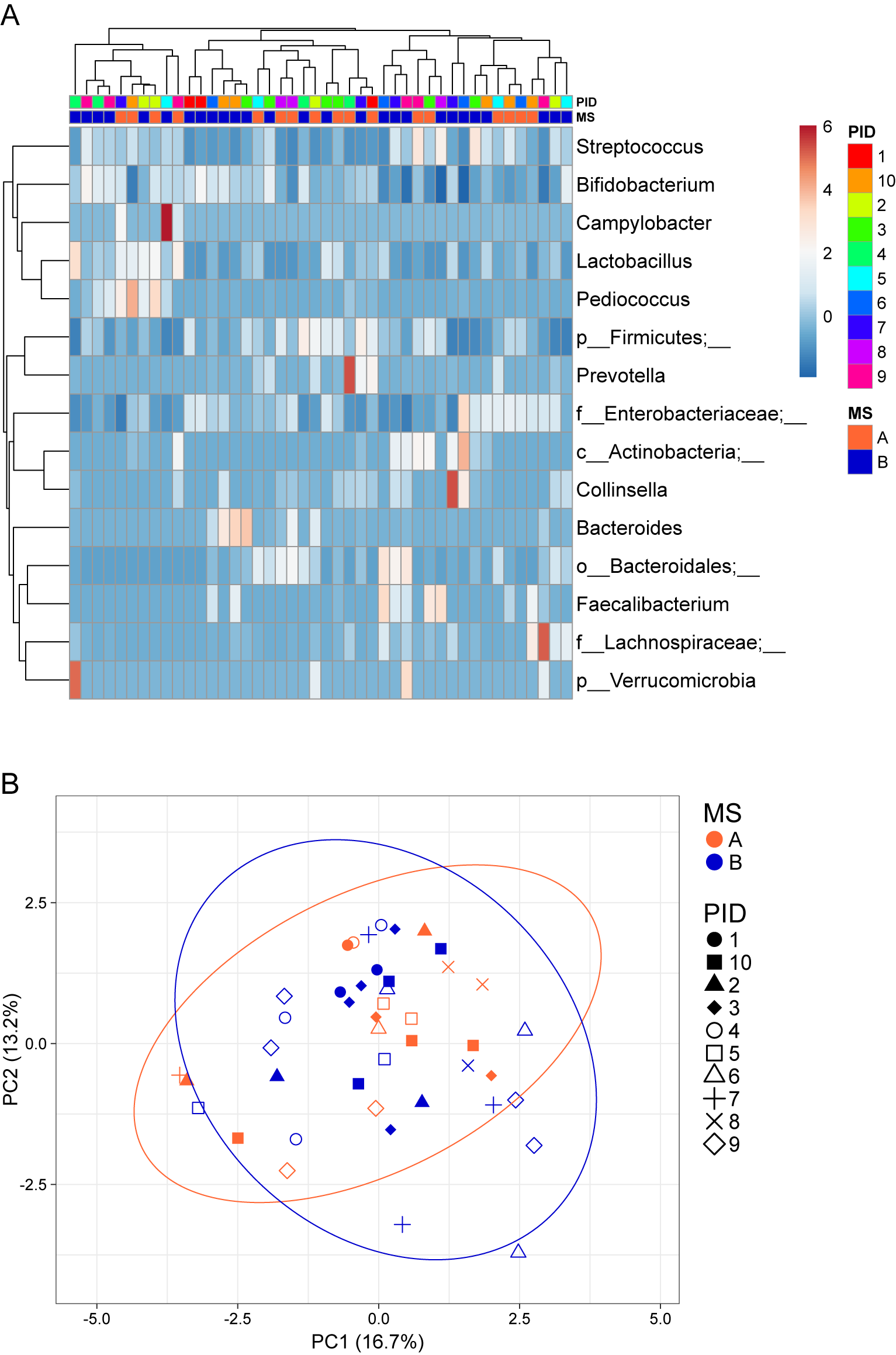

Supplement: jiy740_suppl_Supplementary_Figure_S7 [file jiy740_suppl_supplementary_figure_s7.png]

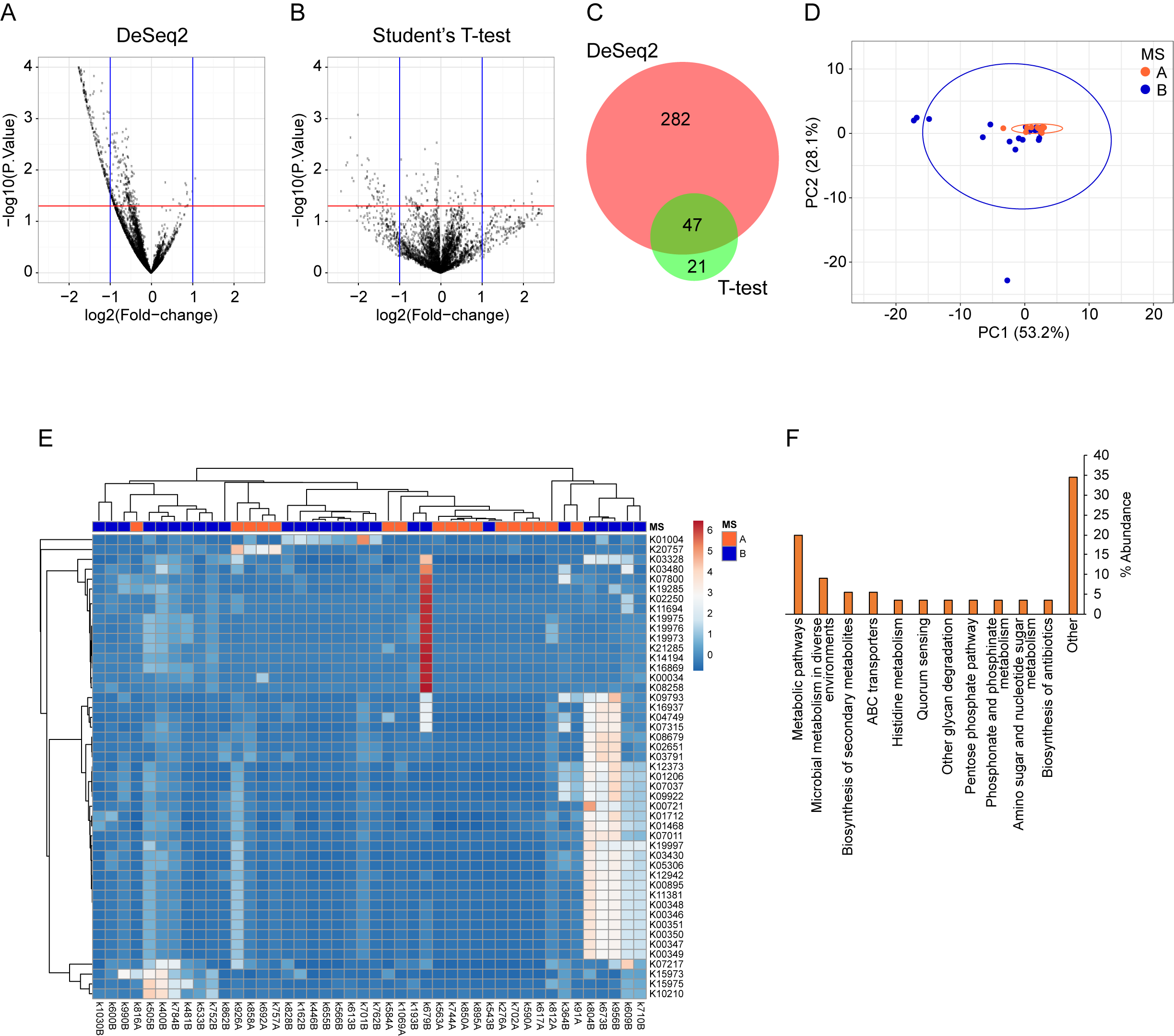

Supplement: jiy740_suppl_Supplementary_Figure_S8 [file jiy740_suppl_supplementary_figure_s8.png]

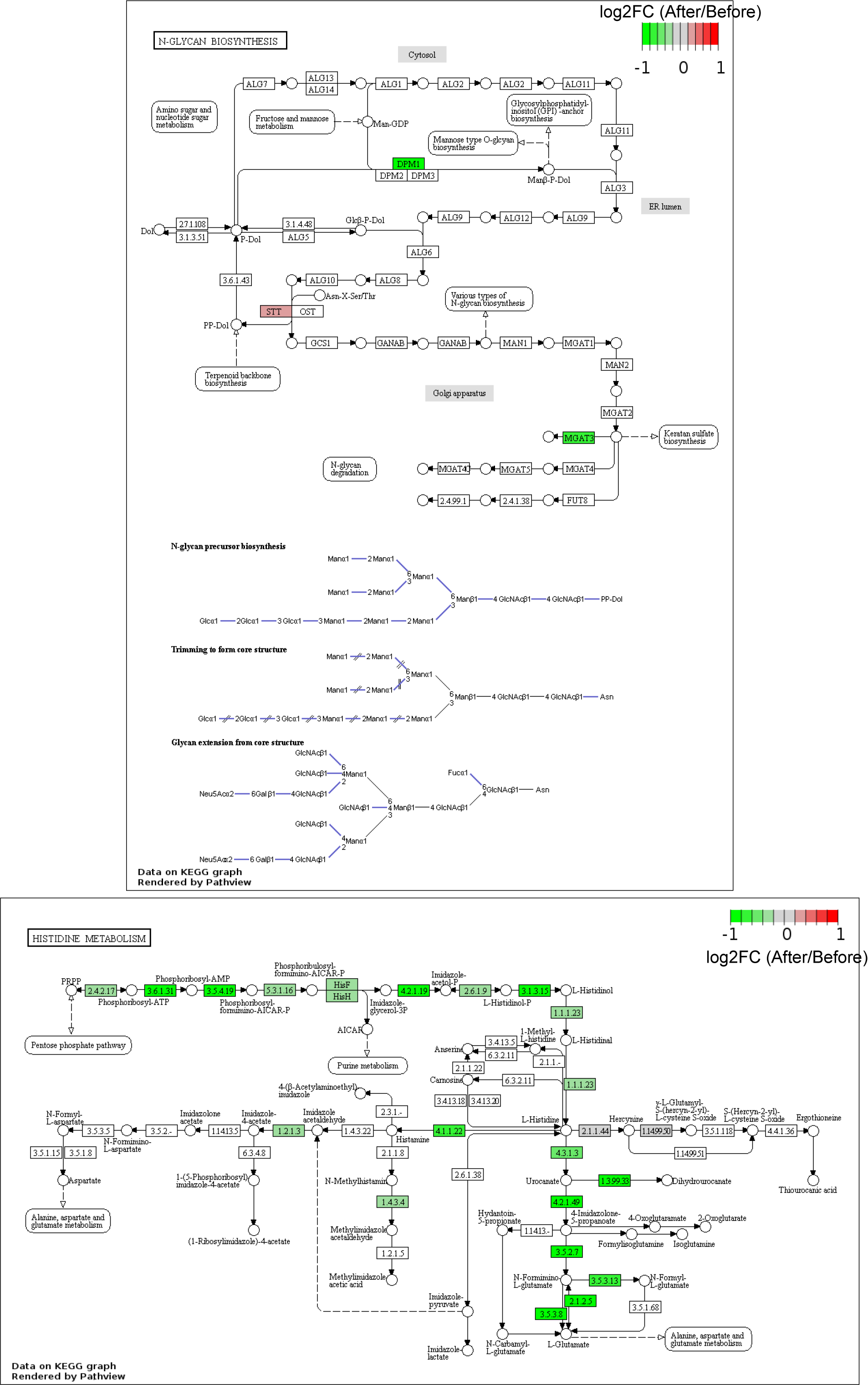

Supplement: jiy740_suppl_Supplementary_Figure_S9 [file jiy740_suppl_supplementary_figure_s9.png]
