# Supplementary material for: Longitudinal Analysis of Infant Stool Bacteria Communities Before and After Acute Febrile Malaria and Artemether-Lumefantrine Treatment
Source: J Infect Dis. 2018 Dec 24;220(4):687–98. doi: 10.1093/infdis/jiy740 (PMC6639600; doi:10.1093/infdis/jiy740)
Supplement: jiy740_suppl_Supplementary_Material [file jiy740_suppl_supplementary_material.pdf]

Longitudinal analysis of infant stool bacteria communities before and after acute febrile malaria and artemether/lumefantrine treatment

Rabindra K. Mandal[1], Rosie J. Crane[2,3]*, James A. Berkley[2,3], Wilson Gumbi[2], Juliana Wambua[2], Joyce Mwongeli Ngoi[2], Francis M. Ndungu[2], and Nathan W. Schmidt[1]*

SUPPLEMENTARY METHODS

Study site and sample collection

Between August 2015 and January 2017, 100 infants resident within one contiguous 26x13km zone comprising 12 villages in Kilifi County, Kenya, were observed from within 14 days of birth until nine months of age. The primary purpose of this cohort study was to describe risk factors for Environmental Enteric Dysfunction. Ethical approval for the cohort study was obtained from KEMRI Scientific & Ethics Review Unit, Kenya (references 2983) and Oxford Tropical Research Ethics Committee, UK (37-15).

Malaria transmission intensity in the area is between moderate and high. Malaria episodes were identified through active and passive case detection. For active detection, participants were visited at home once per week and axillary temperature measured. If fever was present (≥37.5°C), a capillary blood sample was obtained for both rapid diagnostic test (RDT, CareStart kit; AccessBio) immediately at the participant's home, and parasite microscopy later that day at the KEMRI Wellcome Trust Research Programme Clinical Trials Laboratory (KEMRI-WTRP CTL). *Plasmodium* species and density were recorded. Participants with positive RDT and/or slide microscopy were immediately started on a 3-day oral course of AL. If the caretaker reported that the participant had been feverish, but temperature upon home visit

was <37.5°C, repeat temperatures were measured at least twice during the following 24 hours. If at any point the temperature exceeded 37.5°C, blood sampling, and treatment if malaria was detected, was provided as detailed above. Passive case detection occurred in-between weekly home visits when caretakers were encouraged to bring participants to the local primary healthcare facility (Junju dispensary) in the event of fever during working hours, where they would be assessed by the study clinician. Outside of working hours, participants were brought to the home of their local study fieldworker. Antibiotic courses were also prospectively recorded on the study database during weekly home visits by fieldworkers, by the study clinician at Junju dispensary, and for any inpatient admissions by the cohort Principal Investigator (Dr. Rosie Crane) at Kilifi County Hospital.

Stool samples were collected at home every 1-3 weeks by caretakers transferring feces from disposable nappy to a sterile pot using a sterile spatula. Samples were transferred to cold storage (2-8°C) mostly within 1 hour then brought to Junju dispensary where four 0.5-2ml aliquots were created then stored in a dry shipper at -198°C mostly within a further 1 hour. Samples were transported in the dry shipper on a weekly basis to KEMRI-WTRP CTL where they were transferred without thawing to -70°C storage.

Selection of participants and stool samples

Participants were selected for inclusion in this analysis if they had stool samples collected before and after a confirmed clinical malaria episode(s) without antibiotics having been administered between collection time points. Whereas multiple samples were collected for each of these selected participants, only those stool samples meeting the criteria above were selected for analysis, as shown in Figure 1A.

DNA extraction, amplification and sequencing

Stool samples were retrieved from -70°C storage and thawed on ice. From each sample, 200mg was aseptically weighed into a container pre-loaded with ≈370mg of acid-washed 212-300 µM glass beads (Sigma G1277-500G). DNA extraction was then carried out using Qiamp Fast DNA Stool Mini Kit (Qiagen 51604). The manufacturer's protocol was modified in two ways to enhance cell lysis: first by mechanical disruption through bead beating, and second through an additional incubation of the lysate (95°C, 5 minutes) after addition of 1ml inhibitex buffer followed by centrifugation (15000g, 1 minute 30 seconds) to pellet the stool particles. The supernatant was then taken through further steps of inhibitor removal, purification and elution of DNA using spin columns as per kit protocol. DNA yield was determined using Qubit 2.0 Fluorimeter/Qubit dsDNA HS Assay Kit as per protocol.

The V3- V4 hypervariable region of the 16S rRNA gene was targeted for sequencing. Primers targeting this region were constructed with Illumina adapter overhang sequences added to the gene-specific primer sequences. A region of 467 bp was targeted and amplified using the following primers; 16S Amplicon PCR Forward Primer - TCGTCGGCAGCGTCAGATGTGTATAAGAGACAGCCTACGGGNGGCWGCAG and 16S Amplicon PCR Reverse Primer - GTCTCGTGGGCTCGGAGATGTGTATAAGAGACAGGACTACHVGGGTATCTAATCC. Amplifications were done in 25 µl reactions with 12.5 µl Q5® Hot Start High-Fidelity 2X Master Mix (NEB), 1 µl of 1µM forward and reverse 16S Amplicon PCR primer and 2.5 µl of template. The reactions were performed on ABI Veriti thermocyclers (Applied Biosytems) under the following conditions 95°C for 3 minutes, 25 cycles of; 95°C for 30 seconds, 55°C for 30

seconds, 72°C for 30 seconds, followed by 72°C for 5 minutes and a final hold at 4°C. The amplified products were then verified on 1% agarose gel before purifying using Agencourt AMPure XP beads (BeckmanCoulter). Libraries were prepared by ligating Illumina dual indices and Illumina sequencing adapters to the prepared amplicons using the NexteraXT index kit according to the Illumina 16S metagenomic sequencing library preparation protocol (Illumina). Each library was then purified using Agencourt AMPure XP beads (BeckmanCoulter) and thereafter the size and quantity was assessed using the Agilent 2100 Bioanalyzer (Agilent) and the Qubit 2.0 flourimeter (Life Technologies) respectively. The barcoded libraries were pooled at equimolar concentration and 8 pM of the pooled library spiked with 5% Phix (v3) and then sequenced on 300 PE Miseq run using the MiSeq® Reagent Kit v3 (600 cycle). 16S rRNA gene sequences have been deposited in the NCBI short read archive under bioproject XXXXXX (note to editor - to be populated upon acceptance).

Sequence Data analysis

The paired end sequenced 16S rRNA gene (V3-V4) amplicons were analyzed with QIIME 2 (qiime2-2017.8, https://qiime2.org/) using the command line interface. Data were analyzed with three different approaches (Runs). In Run 1, full length forward and reverse reads were used with 140 bp overlapping region. Run 2 had trimmed forward (16 - 290 bp) and reverse reads (1 – 220 bp) based on the Phred quality score with 50 bp overlapping reads. And, Run 3 had high quality forward reads (16 – 212 bp) spanning the complete V3 region of the 16S rRNA gene.

All plugins used for the data analysis were implemented in QIIME2. Raw sequencing data were imported using import plugin, demultiplexed with demux plugin, and sequence quality control and feature table were constructed with DADA2 plugin [1]. DADA2 corrects Illumina

amplicon sequence data where possible, remove phiX reads, and filters chimeric sequences. The resulting feature table were visualized with feature-table plugin and tree for phylogenetic diversity analyses were generated with alignment and phylogeny plugin. Alpha (within group) and beta (between group) diversity analyses were performed using diversity plugin at sampling depth of 30,000 sequences for Run 3 and 5000 sequence depth for Run 2. Principal coordinates analysis (PCoA) plots were made with emperor plugin. Alpha diversity metrics used were OTUs (richness), Shannon index (evenness and abundance), and pielou_e (eveness). Beta diversity metrics were Bray Curtis, weighted UniFrac and unweighted UniFrac distance. Naive Bayes classifier trained the classifier on the Greengenes 13_8 99% OTUs database on the trimmed 16S sequence (V3-V4 region) that was amplified with forward primer (341F: 5'-CCTACGGGNGGCWGCAG-3') and reverse primer (805R: 5'-GACTACHVGGGTATCTAATCC-3') and taxonomic analysis was performed using feature-classifier plugin. OTU interaction map was made using Cytoscape [2] and graphical phylogenetic analysis plot was produced using GraPhlAn [3]. Metagenomic capacity were predicted based on 16S rRNA gene using online tool Piphillin (http://secondgenome.com/solutions/resources/data-analysis-tools/piphillin/) with default settings [4]. Briefly, raw feature table (OTU Abundance Table) and representative sequence (req-seqs) were uploaded and metagenomic capacity were predicted against Kyoto Encyclopedia of Genes and Genomes (KEGG) database (KEGG; May 2017 version). In some cases heatmaps and PCoA plots were drawn with ClustVis (https://biit.cs.ut.ee/clustvis/) [5]. GraphPad software (v 7.0b) was used for statistical analysis and some data display.

Statistical significance for alpha and beta diversity between groups were performed with linear mixed model effects model (LMEM) using QIIME2 longitudinal plugin for longitudinal

analysis. At first, differentially abundant bacterial taxa were screened with linear discriminant analysis (LDA) effect size (LEfSe; http://huttenhower.sph.harvard.edu/galaxy) bioinformatics pipeline [6]. Secondly, the significance level was verified using repeated measures (LMEM). Analysis of differential metabolic pathways and genes before and after malaria episode/AL treatment were performed using RNA-seq 2G online tool (http://52.90.192.24:3838/rnaseq2g/) with default settings using two differential expression (DE) methods (Students T and DESeq2) [7]. Log2FC (fold change) cutoff for differentially expressed KEGG orthologs was ≥ 1 and *P* value ≤ 0.05 for both metabolic pathways and KEGG genes. Analysis includes all stool samples, unless otherwise indicated when paired before and after malaria episode stool samples are compared.

SUPPLEMENTARY TEXT

Forward reads outperformed the joined reads

Demultiplexing the raw sequencing reads from an amplicon spanning V3-V4 of the 16S rRNA gene in all 44 stool samples (Figure 1A), plus an additional 4 samples that were not included in the analysis owing to the identification of an intervening antibiotic treatment, produced an average of 250,169.94 (SE ± 6971.34) and median of 252,590.5 reads per sample (Minimum = 50,800 and Maximum = 335,554) totaling 12,008,157 reads (Supplementary Table 1). The 16S rRNA gene sequencing data were analyzed by three different approaches with varying lengths of forward and reverse reads based on the Phred quality score. In the first approach (Run 1) full length forward and reverse read were used, in the second approach (Run 2) trimmed forward and reverse reads were used, and in the third approach (Run 3) only trimmed high quality forward reads were used (Supplementary Figure 1A-B, see Materials and Methods for more detail). After

the quality control steps using DADA2, Run 1 had very few reads (11.81 ± 2.54 per sample and were not considered for downstream analysis), Run 2 had intermediate reads (28,203 ± 1,225 per sample), while Run 3 had the highest reads (163,811 ± 4,622 per sample) that were assigned taxonomically (Supplementary Figure 1C). Run 3 had significantly higher confidence (0.91 ± 0.002) for taxonomically classified reads than Run 2 (0.88 ± 0.003) ($P < 0.0001$, Mann-Whitney test, Supplementary Figure 1D). Consistent with the differential confidence in classification, Run 2 had a different profile than Run 3 at the phylum level (Supplementary Figure 1E-F), the ratio of Firmicutes to Bacteroidetes was two times higher in Run 2 (9.52) than Run3 (4.72), and most notably, Run 2 had a significantly higher portion of reads (16.97 ± 1.9 % per sample) classified only to kingdom level (bacteria) than Run 3 (0.59 ± 0.1 %) ($P < 0.0001$, Mann-Whitney test, Supplementary Figure 1E-F). Furthermore, there was a significantly higher number of Observed_OTUs in Run 3 (104.1 ± 3.93) than Run 2 (62.81 ± 3.2) ($P < 0.0001$, Mann-Whitney test, Supplementary Figure 1G). Consistent with the increased diversity of taxonomically assigned reads in Run 3 (Supplementary Figure 1F) compared to Run 2 (Supplementary Figure 1E), the Shannon index and pielou_e were significantly lower in Run 3 compared to Run 2 ($P < 0.0001$, Mann-Whitney test, Supplementary Figure 1H-I). Overall, Run 3 outperformed Run 2 with regards to Phred quality score, confidence in taxonomically classified reads and alpha diversity. Error prone sequence correction and comparatively lower quality sequence reads at 3' end of reads, a limitation of most DNA sequencers, might have contributed to the poor performance of stitched reads (Run 2). Consequently, Run 3 was used for all the analysis performed in this study.

SUPPLEMENTARY TABLES

Supplementary Table 1. Metadata file with read numbers and alpha diversity indices.

Supplementary Table 2. Antibiotic courses.

Supplementary Table 3. Malaria episode characteristics.

Supplementary Table 4. Alpha diversity analysis.

SUPPLEMENTARY FIGURES

Supplementary Figure 1 Quality analysis of 16S rRNA gene sequencing. V3 and V4 region were subjected to MiSeq sequencing. Sequence quality control and feature table were constructed with DADA2 implemented inside QIIME2. Three runs were performed with varying read length depending on the Phred quality score. Run 1: Full length forward and reverse reads with 140 bp overlapping region. Run2: Forward reads (16 – 290 bp) and reverse reads (0 – 220) were trimmed for low quality reads conserving 50 bp overlapping reads. Run 3: High quality forward reads (16 – 212 bp). A and B) Overall Phred score of forward and reverse read respectively. C) Reads taxonomically classified by DADA2 with three different runs. D) Confidence of reads classified by DADA2. Box denotes 25th and 75th percentile with median in between and whisker denotes lowest and highest value. Data were analyzed by the Mann-Whitney test. E and F) Taxonomic classification at phylum level. Alpha diversity of Run 2 and Run 3 indicated by Observed_OTUs (G), Shannon Index (abundance + evenness) (H) and pielou_e (evenness) (I). G-I) Individual samples from each run are shown along with the mean ± S.E. Data were analyzed by the Mann-Whitney test.

Supplementary Figure 2 OTU interaction maps show no distinct pattern between paired before and after malaria episode/AL treatment samples. A) Interaction between 24 paired samples

(Figure 1) and 997 sequence variants (SV). Nodes (SVs) shared by the most samples are placed at the core of map as indicated by edge length from the samples. B) Overall top 300 SVs shared between the before and after malaria episode/AL treatment stool samples in Kenyan infants which represents the core of interaction map. C) Relative abundance of sequence variants shown in B and the remaining 697 SVs.

Supplementary Figure 3 No effect of infant age on alpha diversity. Pearson correlation of age versus Observed_OTUS (A,C) and age versus Shannon Index (B,D) in overall 44 stool samples (A,B) and infants that had not and had antibiotics course (C,D).

Supplementary Figure 4 Phylogenetic graph shows no obvious difference in the microbiota taxonomic composition between before and after malaria episode/AL treatment. Taxonomic cladogram represents the phylogenetic analysis before malaria/AL treatment (A), after malaria/AL treatment (B), and combined before and after malaria episodes/AL treatment (C). Size of nodes correlates with their relative abundance and different colors indicate different clades. The graph was produced using Graphical Phylogenetic Analysis (GraPhlAn) tool.

Supplementary Figure 5 Minimal taxonomic difference between before and after Malaria/AL treatment. A) Cladogram showing discriminant features at kingdom, phylum, class, order, family and genus level. Rings are arranged according to the taxonomic level. Outermost- Genus and inner most –Kingdom. B) Bar graph shows the fold change of differentially abundant features. Alpha value for the pairwise Wilcoxon test during LEfSe analysis was set to 0.05. Threshold on

the logarithmic LDS score for discriminative features were set to 2 and 0.2. C and D). Differentially abundant features re-evaluated using LMEM.

Supplementary Figure 6 Taxonomic assignment at genus level shows no distinct clustering between the before and after malaria episode/AL treatment stool samples. A) Heat map of overall top 15 genera. B) PCoA plot based on top 15 genera. MS: Malaria status; B- Before malaria; A- After malaria; PID: Participant ID.

Supplementary Figure 7 Linear mixed effect model analysis of sequence variants identified by LEfSe analysis (see Figure 5). A) LMEM analysis of sequence variants (SV38 and SV43) that were identified as being overly abundant in before malaria/AL stool samples. B) LMEM analysis of sequence variants (SV29 and SV72) that were identified as being overly abundant in after malaria/AL stool samples.

Supplementary Figure 8 Predicted metagenomic capacity at KEGG ortholog (KO) level identifies minimal differences between the before and after malaria episode/AL treatment stool samples. Volcano plot of KO with DeSeq2 (A) and student's T-test (B). C) Overlapping KO identified by DeSeq2 and T-test. D) PCoA plot and E) Heat map and F) Pathway enrichment of the 47 overlapping KOs identified by DeSeq2 and T-test.

Supplementary Figure 9 KEGG orthologs (KOs) of the A) N-Glycan biosynthesis pathway and B) Histidine metabolism pathway having differential abundance in the before and after malaria episode/AL treatment stool samples (see Figure 6 and Supplementary Figure 8). The predicted

KOs are visualized on KEGG pathway constructed using Pathview. The annotation for the graph are same as the online source (https://pathview.uncc.edu/overview#kegg_view).

SUPPLEMENTARY REFERENCES

1. Callahan BJ, McMurdie PJ, Rosen MJ, Han AW, Johnson AJA, Holmes SP. DADA2: high-resolution sample inference from Illumina amplicon data. Nature methods **2016**; 13(7): 581-3.
2. Shannon P, Markiel A, Ozier O, et al. Cytoscape: a software environment for integrated models of biomolecular interaction networks. Genome research **2003**; 13(11): 2498-504.
3. Asnicar F, Weingart G, Tickle TL, Huttenhower C, Segata N. Compact graphical representation of phylogenetic data and metadata with GraPhlAn. PeerJ **2015**; 3: e1029.
4. Iwai S, Weinmaier T, Schmidt BL, et al. Piphillin: Improved Prediction of Metagenomic Content by Direct Inference from Human Microbiomes. PloS one **2016**; 11(11): e0166104.
5. Metsalu T, Vilo J. ClustVis: a web tool for visualizing clustering of multivariate data using Principal Component Analysis and heatmap. Nucleic acids research **2015**; 43(W1): W566-W70.
6. Segata N, Izard J, Waldron L, et al. Metagenomic biomarker discovery and explanation. Genome biology **2011**; 12(6): R60.
7. Zhang Z, Zhang Y, Evans P, Chinwalla A, Taylor D. RNA-Seq 2G: Online Analysis Of Differential Gene Expression With Comprehensive Options Of Statistical Methods. bioRxiv **2017**: 122747.
